# A Baseline for Skeletal Investigations in Medaka (*Oryzias latipes*): The Effects of Rearing Density on the Postcranial Phenotype

**DOI:** 10.3389/fendo.2022.893699

**Published:** 2022-06-30

**Authors:** Claudia Di Biagio, Zachary Dellacqua, Arianna Martini, Ann Huysseune, Michele Scardi, Paul Eckhard Witten, Clara Boglione

**Affiliations:** ^1^ PhD Program in Evolutionary Biology and Ecology, Department of Biology, University of Rome ‘Tor Vergata’, Rome, Italy; ^2^ Laboratory of Evolutionary Developmental Biology, Gent University, Department of Biology, Gent, Belgium; ^3^ Aquaculture Research Group (GIA), Universidad de Las Palmas de Gran Canaria, Institute of Sustainable Aquaculture and Marine Ecosystems (ECOAQUA), Las Palmas, Spain; ^4^ Laboratory of Experimental Ecology and Aquaculture, University of Rome ‘Tor Vergata’, Department of Biology, Rome, Italy

**Keywords:** skeleton, plasticity, vertebral body fusion, skeletal anomalies, meristic counts, mineralization

## Abstract

*Oryzias latipes* is increasingly used as a model in biomedical skeletal research. The standard approach is to generate genetic variants with particular skeletal phenotypes which resemble skeletal diseases in humans. The proper diagnosis of skeletal variation is key for this type of research. However, even laboratory rearing conditions can alter skeletal phenotypes. The subject of this study is the link between skeletal phenotypes and rearing conditions. Thus, wildtype medaka were reared from hatching to an early juvenile stage at low (LD: 5 individuals/L), medium (MD: 15 individuals/L), and high (HD: 45 individuals/L) densities. The objectives of the study are: (I) provide a comprehensive overview of the postcranial skeletal elements in medaka; (II) evaluate the effects of rearing density on specific meristic counts and on the variability in type and incidence of skeletal anomalies; (III) define the best laboratory settings to obtain a skeletal reference for a sound evaluation of future experimental conditions; (IV) contribute to elucidating the structural and cellular changes related to the onset of skeletal anomalies. The results from this study reveal that rearing densities greater than 5 medaka/L reduce the animals’ growth. This reduction is related to decreased mineralization of dermal (fin rays) and perichondral (fin supporting elements) bone. Furthermore, high density increases anomalies affecting the caudal fin endoskeleton and dermal rays, and the preural vertebral centra. A series of static observations on Alizarin red S whole mount-stained preural fusions provide insights into the etiology of centra fusion. The fusion of preural centra involves the ectopic formation of bony bridges over the intact intervertebral ligament. An apparent consequence is the degradation of the intervertebral ligaments and the remodeling and reshaping of the fused vertebral centra into a biconoid-shaped centrum. From this study it can be concluded that it is paramount to take into account the rearing conditions, natural variability, skeletal phenotypic plasticity, and the genetic background along with species-specific peculiarities when screening for skeletal phenotypes of mutant or wildtype medaka.

## Introduction

Small teleost fish such as *Danio rerio* (zebrafish) and *Oryzias latipes* (medaka) are widely used as models for human skeletal diseases (1). Basic pathways of endoskeletal development and mineralization are highly conserved between mammals (i.e., humans) and teleost fish (1, 2) including molecular profiles of bone-inducing and regulating factors (3). Medaka belongs to the family of Adrianichthyidae (4) and has been used as a model species in Asia since the beginning of the 20^th^ century (5–7). Medaka differs from zebrafish in several key features. Firstly, medaka are characterized by a longer ontogenetic period: embryos hatch after 7-9 days (26°C), whereas in zebrafish hatching occurs after 2-3 days (28°C) (8). Medaka can also withstand a wider temperature range, which can be employed in laboratory conditions to slow down the speed of development (9, 10). Fittingly, as descendant of marine ancestors, medaka is an euryhaline species, thus adults are capable of surviving in both limnic and marine conditions (11). As typically found in advanced teleosts, medaka’s genome is small: with 700 Mb (12, 13), it represents about half the size of the zebrafish genome. The large availability of inbred medaka strains enables effective genetic screening and mutagenesis mapping. Another advantage of using medaka as a model organism is the physical transparency of the individuals up to the early juvenile stages: this facilitates visualization of vertebral column elements and rapid screening for defects over a long period of skeletogenesis. In contrast to zebrafish, which shares cellular bone with basal Osteichthyes and tetrapods, including mammals, medaka’s bone is void of osteocytes (acellular bone), a characteristic of advanced teleosts. Nonetheless, bony structures are remodeled and respond to mechanical loading (14–16). The macro and nanostructure of the vertebral bodies as well as their mechanical properties are well conserved and comparable between zebrafish and medaka (17). In both species, and in contrast to mammals with a long intrauterine development, teleosts hatch as embryos (18) and skeletal development continues after hatching, subjected to the influence of multiple biotic and abiotic variables. Thus, the skeletal phenotype is substantially and continuously influenced by environmental factors that complement the genetic background (19, 20). There is comprehensive literature on the effects of the environment on the skeletal health of marine and freshwater teleosts. Rearing density has been described as an environmental variable that influences skeletal development in farmed fish and high densities have been reported as driving factors that induce skeletal anomalies. So far, relatively little attention has been given to study the effects of rearing density on model fish species held in laboratory conditions. For zebrafish, however, recent studies on wildtype (WT) animals have shown that high rearing density results in reduced size, increased variation of skeletal characters and skeletal anomalies in adults (21). Although variation is an intrinsic characteristic of biological entities, reliable wildtype individuals are a fundamental prerequisite for employing model organisms in biological investigations. When screening for mutant phenotypes or evaluating the effects of experimental variables, it is fundamental to discern between natural variation, unaccounted parameters and variations induced by the tested conditions. Textbooks report a thorough description of rearing techniques, maintenance, and rearing density requirements for different medaka life stages (9). Meristic counts of postcranial skeletal elements have been published for wild strains (22–24). For laboratory strains (or domestic/aquarium stocks), counts are available for vertebral centra and dermal fin rays (25–28), however the occurrence of density-dependent variations and anomalies and the extent of their prevalence has not yet been investigated. Given the growing utilization of juvenile and adult medaka in experiments, the aim of this paper is to provide baseline information for the postcranial skeleton based on an evaluation system that has been successfully applied to zebrafish (21, 29). For the purpose of this study, WT medaka were reared from hatching up to 40 days (hereafter dph) in a closed system with recirculating water at three different densities and the effects on survivorship, size, meristic counts, mineralization of skeletal elements, and variation in number and shape of elements are described. Deeper insights on cellular alterations, mineralization patterns, and occurrences of bone remodeling in the fusion of preural centra are reported.

## Materials and Methods

### Ethical Statement

The animal study was reviewed and approved by the Italian Ministry of Health. The approval (N° 133/2021-PR) was issued pursuant to the Italian art. 31 of Legislative Decree 26/2014 and follows the Italian and European regulations.

### Testing the Effect of Stocking Density: Experimental Rearing

The experimental rearing was carried out at the Experimental Biology and Aquaculture Laboratory, University of Rome ‘Tor Vergata’, Italy. The water for the experimental rearing was processed with an osmotic filtering system (Askoll, 4 Stages Pro System 75GPD), supplemented with salts (0.005% Sera Mineral Salts, 1 mM NaHCO_3_) and UV sterilized (AQL, External Sterilizer Pro 18W*)*. The aquaria shared water from a single centralized recirculating system provided with a heater (Eheim-Jager, ThermoControl) mechanical (Askoll Partiko), chemical (Askoll Adsorbor, active carbon), biological (Askoll Puremax), and UV filters to maintain constant temperature (26°C), pH (7.2-7.5), oxygen (> 90%), connectivity (300-500 µS) and reduce the level of ammonia. These controls were implemented in order to standardize identical rearing conditions in all of the aquaria, except for the stocking density. The photoperiod was 12:12 light-dark cycle. The levels of O_2_, nitrites, nitrates, ammonia, pH, and water hardness were checked weekly (Hanna, HI9829, Sera NH3/NH4-Test and Sera Quick Test 6 in 1). Once a week, 1/3 of the recirculating water was renewed. Medaka of the CAB line were reared according to standard procedures (9, 30). The broodstock was maintained in 4 separate 3L tanks, each containing 5 females and 2 males, for a total of 20 females and 8 males. Egg spawning was induced by switching the light-dark cycle to 14:10. Eggs were collected from the belly of each female, transferred altogether to a petri dish and gently separated with tweezers under a stereomicroscope with cold light. The vital eggs were incubated in a thermostatic chamber at 26°C in 500 mL of filtered freshwater supplemented with 0.0002% methylene blue until hatching, at a density of 150-200 eggs/500 mL with a 12:12 light-dark photoperiod. To test the effects of rearing density, medaka from the same spawning event which hatched on the same day were utilized. To avoid any undesirable selection, the hatched medakas were collected in a 200 mL beaker with 30 mL water, mixed altogether and randomly transferred into 3L aquaria to obtain the following densities (**Table 1**): (1) LD or low density: 5 fish/L in 4 aquaria (total of 60 medaka); (2) MD or medium density: 15 fish/L in 1 aquarium (total of 45 medaka); (3) HD or high density: 45 fish/L in 1 aquarium (total of 135 medaka). The rationale underlying the choice of these experimental densities is based on guidelines available for this species (9). For 3L tanks, Kinoshita and colleagues suggest 16 medaka/L for rearing fish up to 30 dph; from 30-60 dph they suggest reducing the density to 10 medaka/L. Therefore, because individuals were reared from hatching up to 40 dph, an intermediate density of 15 medaka/L was chosen as a control and is referred to as medium density (MD). We tested a lower and a higher density by a factor of three. The fish were fed three times per day with a commercial diet ZEBRAFEED^®^ (Sparos, Portugal), with increasing granulometry according to the size of fish, as indicated in the manufacturer’s instructions. At the end of the experimental rearing (40 dph), all the juveniles were anaesthetized with tricaine (Sigma Aldrich) according to their size (MS-222 80-120 mg/L) and imaged with an Axiozoom V.16 camera (Zeiss, Germany). 3 HD individuals with vertebral body fusions and 3 controls were euthanized with a tricaine overdose (MS-222, 300 mg/L), fixed in 4% paraformaldehyde (PFA)/1.5% glutaraldehyde (GA)/0.1 M sodium cacodylate buffer (pH 7.4)/0.001% CaCl_2_ and utilized for histological and enzyme histochemical analyses. The other individuals were euthanized, fixed in 4% PFA/1.5% GA overnight at 4°C and transferred through increasing ethanol concentrations up to 70% ethanol for anatomical inspections.

**Table 1:** Rearing parameters.

Condition	Initial density (medaka/L)	Initial number (medaka/3L tank)	Survivorship(%)	Final density (medaka/L)	Final number (medaka/3L tank)
**LD**	5	15	91 (87, 93, 93, 93)	5 (4, 5, 5, 5)	14 (13, 14, 14, 14)
**MD**	15	45	98	15	44
**HD**	45	135	94	42	127

Initial and final number (medaka/3L tank) of individuals and density (medaka/L), and survivorship at 40 dph are reported for the tested densities: low (LD), medium (MD) and high (HD). For the LD group, data represents the average of the 4 LD tanks. The individual values of each tank are reported in parentheses.

### Terminology

**Figure 1:**
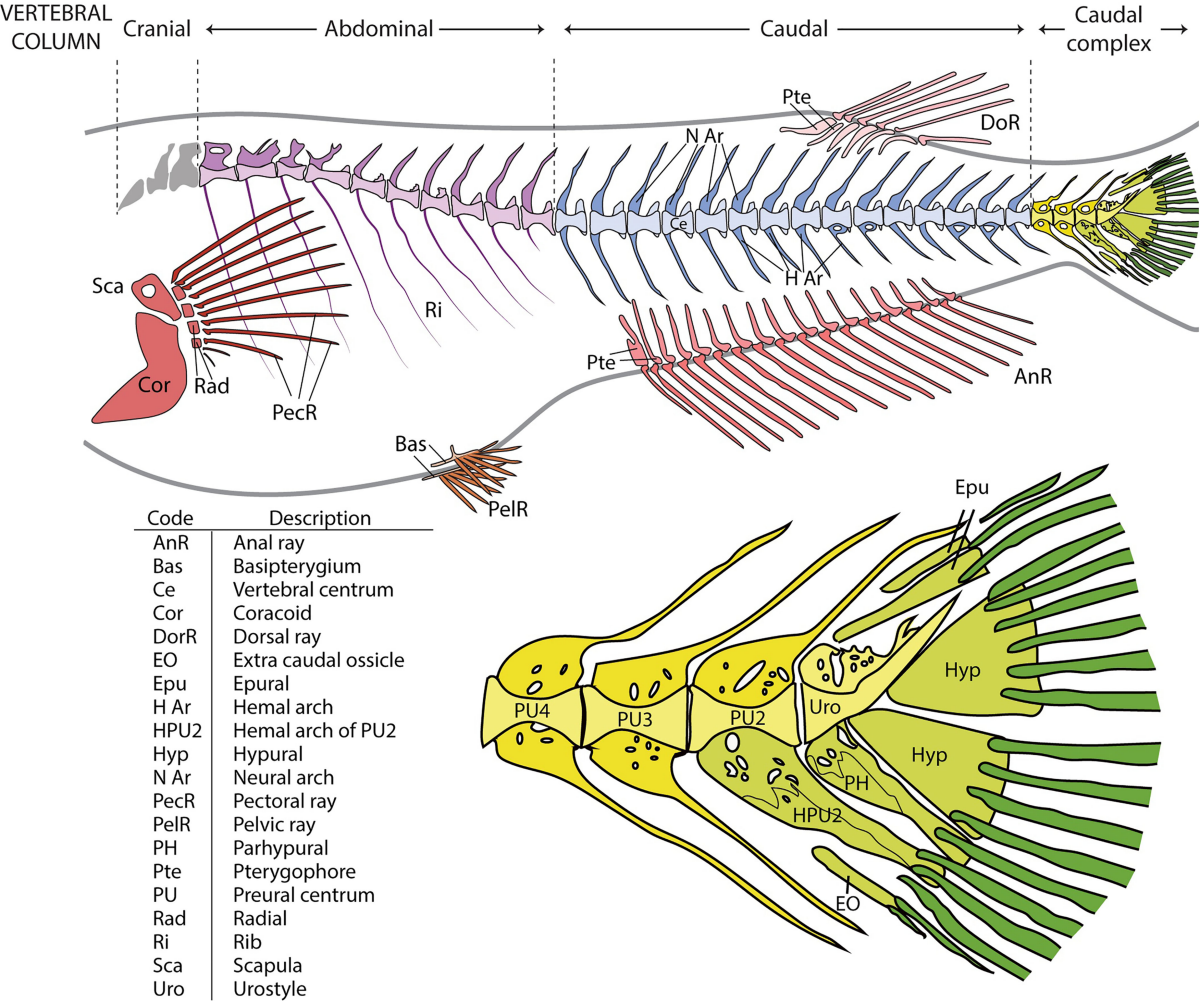
Overview of the vertebral column and fins in medaka and close-up on the caudal complex skeleton. Medaka have two paired fins (pectoral fin: red; pelvic fin: orange) and three unpaired fins (anal fin: dark pink; dorsal fin: light pink; caudal fin: green). The vertebral column is divided into 4 regions: cranial (grey), abdominal (purple), caudal (blue), and caudal complex (yellow). The vertebral bodies are shown in a lighter tone, the neural and hemal arches in a darker tone. Similarly, the internal supports of the paired and unpaired fins (radials, scapula and coracoid of the pectoral fin, basipterygia of the pelvic fin, and pterygophores of the dorsal and anal fins) have a lighter tone, whereas the dermal fin rays a darker tone. In the bottom picture, the caudal complex vertebrae (in yellow) consist of 3 preural vertebrae (PU). The modified hemal arch (parhypural, PH) of the urostyle (Uro) and the modified hemal arch of PU2 (HPU2) are classified as part of the caudal fin, together with rays, epurals (Epu), hypurals (Hyp), and the extra caudal ossicle (EO). The PH and HPU2 have been modified to increase the stiffness of the fin and to support the caudal rays (dark green). The table represents an overview of all the investigated skeletal elements.

In line with the terms used in comparative vertebrate anatomy (31) and enabling cross-comparisons with studies carried out on fish models, such as zebrafish, the axial skeleton was subdivided into cranial, abdominal, caudal, and caudal complex vertebrae (**Figure 1**). The cranial vertebrae, which are characterized by the absence of ribs, were not clearly discernable in all the specimens and were therefore not considered in the data analyses. The term abdominal is used to indicate vertebrae that carry ribs and/or open hemal arches (without hemal spines). The term caudal follows previously published studies on model fish, i.e., zebrafish. Concerning the caudal complex, the terminology we used is in accordance with Arratia and Schultze (32) and Bensimon-Brito et al. (33), and revised by Wiley et al. (34) in which preural centra are terminal vertebrae supporting the caudal fin and carrying modified hemal and neural arches; ural centra carry hypurals and epurals and the urostyle is the compound element of the vertebral column composed of preural 1 and all of the urals (32–36). As far as the caudal fin elements are concerned, we used the same terminology that is used for zebrafish which also applies to medaka (taking into consideration the different counts of hypurals and epurals) (34, 37). Epurals, hypurals, parhypural, modified hemal spine of PU2 (HPU2), and the extra caudal ossicle (EO) are endoskeletal elements that support the caudal fin. The underlining basis of the choice to include them among the caudal fin elements takes into consideration that their modified shape is a functional characteristic which support the principal caudal rays and could lead to increasing the stiffness of the caudal fin. This inclusion is in accordance with the other fin elements, which are made up of dermal rays and endoskeletal support (i.e., pterygophore, radials, basipterygia). The term ‘malformation’ refers to a morphological defect occurring during development. It is a broad term including congenital malformations as well as malformations with different etiologies, including environmental factors. The term ‘anomalies’ refers to both the defects that could be classified as malformations and natural variations which cannot be ascribed to a specific causative factor (38).

### Anatomical Survey: Meristic Counts, Mineralization State, Skeletal Anomalies

The fixed individuals were whole mount-stained with Alizarin red S (ARS), according to Taylor and Van Dyke (39). To properly visualize the vertebral column and the fin endoskeletal elements, the scales were completely removed. Imaging was performed with the Axiozoom V.16 stereomicroscope (Zeiss, Germany) equipped with a 5MP CCD camera. The standard length (SL) of all individuals was recorded on stained samples with Image J (Fiji, version 1.51), by measuring the distance between the tip of the upper jaw (premaxillary) and the insertion of the caudal fin rays. To investigate a potential effect of rearing density on skeletogenesis, the mineralization of all skeletal elements was examined. Caudal fin rays were considered fully mineralized if at least two segments were detected in at least 5 superior and 6 inferior dermal rays. This assumption was necessary considering the early juvenile stage. The frequencies (%) of individuals with fully, partially-, and non-mineralized skeletal elements were reported for all individuals belonging to the three density groups. The same analysis was performed only on individuals of the same size class SL: 11.5-15.5 mm (38 individuals for the LD group, 37 MD, and 63 HD). To statistically evaluate the link between the mineralization state and length, a logistic regression analysis was performed. For each skeletal region (i.e., vertebrae, fin rays, pterygophores), the score of 0 was assigned to the non- and/or partially mineralized elements, whereas a score of 1 was assigned to fully mineralized elements. The monitoring of the skeletal anomalies was carried out on the base of the alpha-numeric classification proposed by Martini *et al.* (21) for zebrafish and adapted to medaka (**Supplementary Table 1**). The vertebral region A in medaka refers to the cranial vertebrae and not to the Weberian region (21), which is absent in medaka. It was decided to maintain the same terminology enabling a more efficient comparison between the two widely used fish models. The capital letter indicates the skeletal region, the number corresponds to a skeletal element, and the letter code specifies the type of anomaly. Skeletal anomaly data were expressed in a raw matrix (RM) and used to calculate the frequencies (%) of each type of anomaly over the total number of anomalies, in each group. The RM was subsequently transformed into a binary matrix (BM) which was used to calculate the prevalence of individuals affected by each anomaly type. The following descriptive metrics were calculated, for each group: 1) relative frequency (%) of individuals with at least one anomaly; 2) malformation index, i.e., the average anomalies’ load (number of total anomalies over number of individuals with at least 1 anomaly); 3) relative frequency (%) of individuals with axis and/or vertebral body anomalies. The frequencies (%) of individuals affected by each type of anomaly over the total number of individuals are reported in **Supplementary Table 2**. The axis or vertebral centra anomalies reported in **Table 2** include both extended and localized deviations of the axis as well as variations in shape/size of vertebral centra (see **Supplementary Table 2** for a comprehensive list of the considered anomalies).

**Table 2:** Descriptive skeletal metrics of the experimental groups.

	LD	MD	HD
**Number of observed individuals**	55	44	121
**Frequency (%) of individuals with at least one anomaly**	95^a^	98^a^	97^a^
**Malformation index**	5	5	5
**Frequency (%) of individuals with axis deviations and/or vertebral centra anomalies**	45^a^	59^a,b^	69^b^

The numbers are reported for each experimental group (LD, MD, HD). The malformation index refers to the average number of anomalies per observed individual. “N-1” *chi-squared* test for proportions, followed by Bonferroni correction. Different letters (a, b) indicate statistically significant difference among groups (p <0.05).

### Statistics

Differences between median SL values at different rearing densities were analyzed by means of a Kruskal-Wallis test, with *a posteriori* pairwise Mann-Whitney tests and Bonferroni correction. Concerning the skeletal anomalies, statistical differences in proportions were analyzed by the “N-1” *chi-squared* test as recommended by Campbell (40) and Richardson (41) with the MedCalc software, followed by the Bonferroni correction for multiple testing. The confidence interval was calculated according to the recommended method given by Altman *et al.* (42). The independence between variables (i.e. vertebral numbers and rearing density or skeletal anomalies and rearing density) was tested with a *chi-squared* test, followed by the Bonferroni correction for multiple testing. The χ^2^ components were calculated to determine which frequencies were significantly higher or lower than the expected values. For what concerns the mineralization data, logistic regression was calculated for each skeletal element and grouped by rearing density (LD, MD, and HD). The logistic function represents the probability (between 0 and 1) that a skeletal element is mineralized for a specific standard length. To obtain a linear relationship between SL and mineralization, the probability was transformed into the log odds of obtaining a fully mineralized skeletal element. To test whether the slope is significantly different from 0 (odds ratio ≠ 0), the Wald test and the likelihood ratio test were performed. All the statistical analyses except those about differences between proportions were performed with Past V 4.01 (43).

### Histological Studies

The specimens employed for histological analyses were incubated in fixative for 2 hours, rinsed in PBS and decalcified (when necessary) in 10% EDTA/4% PFA at 4°C. Dehydration and embedding in glycol methacrylate was performed according to Witten *et al.* (44). In brief, the juveniles were rinsed with PBS, dehydrated with a graded series of acetone (30, 45–48) and stored at -20°C until embedding. Samples were pre-impregnated in monomer solution ((2-hydroxyethyl)-methacrylate, ethylene glycol monobuthyl ether, benzoyl peroxide) for 1 h on ice and transferred into fresh solution for 24 h at 4°C. The monomer solution was supplemented with 2% catalyst (N,N-dimethylaniline, poly-ethylenglycole-200) for embedding, which was incubated for 48 h at 4°C. The polymerization completes in the following 24 h at RT. 3 µm sections were cut on an automated microtome Microm HM 360 (Marshall Scientific, Hampton, NH, USA). Toluidine blue and Verhoeff Elastin staining were performed according to Humason et al. (49).

### Enzyme Histochemical Procedures: Measuring TRAP and ALP Activity

Specimens used for the demonstration of tartrate-resistant acid phosphatase (TRAP) and alkaline phosphatase (ALP) activity were fixed, decalcified (only for TRAP), dehydrated and embedded as previously described. 4 µm sections were cut on an automated microtome Microm HM 360 (Marshall Scientific, Hampton, NH, USA). Demonstration of TRAP was performed according to Witten et al. (44) and Nemoto et al. (50). The ALP staining was based on the azo-dye-coupling method (51, 52) and performed according to Witten and Villwock (53).

## Results

### Survivorship and Standard Length

**Figure 2:**
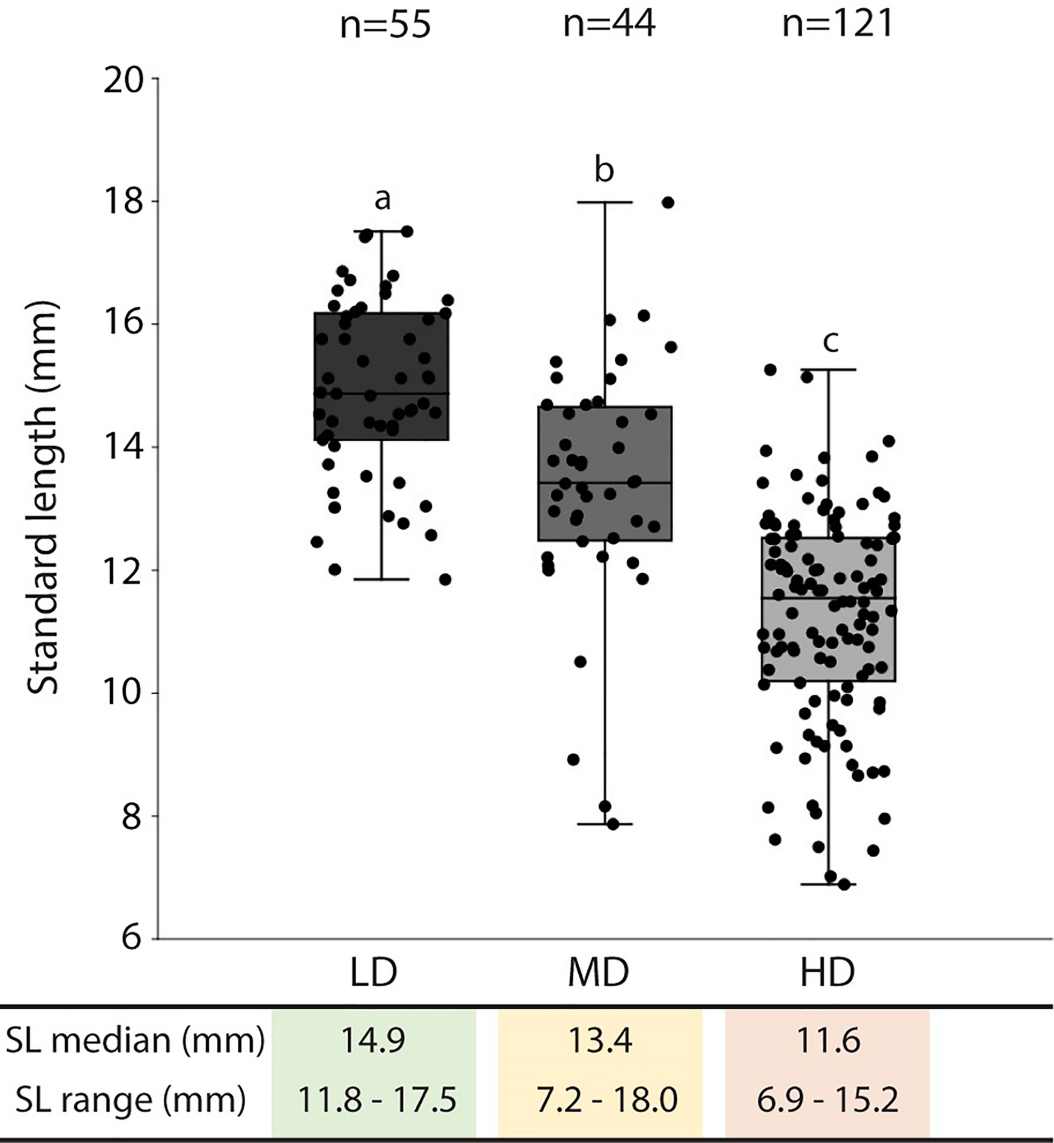
Standard length in medaka reared at different densities. The standard length (SL: mm) median and range are presented in the table below the graph. The SL values are reported with box & whisker plots: the midline in each box is indicative of the median, whereas min and max values are shown with whiskers. The box represents the central percentile. Different letters indicate statistically significant differences among groups (Kruskall-Wallis test, followed by *a posteriori* pairwise Mann-Whitney tests with Bonferroni correction, p <0.0001). SL, standard length (mm); n, number of samples; LD, low density; MD, medium density; HD, high density.

The average survivorship of the individuals reared at low (LD), medium (MD), and high (HD) density conditions at 40 dph is 91%, 98%, and 94% respectively (**Table 1**). Due to the 6% mortality in the HD aquarium, the final density in this group was reduced to 42 individuals per liter. The density in the MD group remained unchanged, 15 individuals/L. For LD, the final density was maintained (5 individuals/L) in three of the aquaria replicates and was reduced to 4 individuals/L in one aquarium. Rearing density is found to be negatively related to the standard length (SL) of the animals. As shown in **Figure 2**, the SL of HD individuals (6.9-15.2 mm; median: 11.6 mm) is significantly lower compared to MD individuals (7.2-18 mm; median: 13.4 mm), and SL in MD individuals is significantly lower compared to LD individuals (11.8-17.5 mm; median: 14.9 mm).

### The Postcranial Skeleton

**Figure 3:**
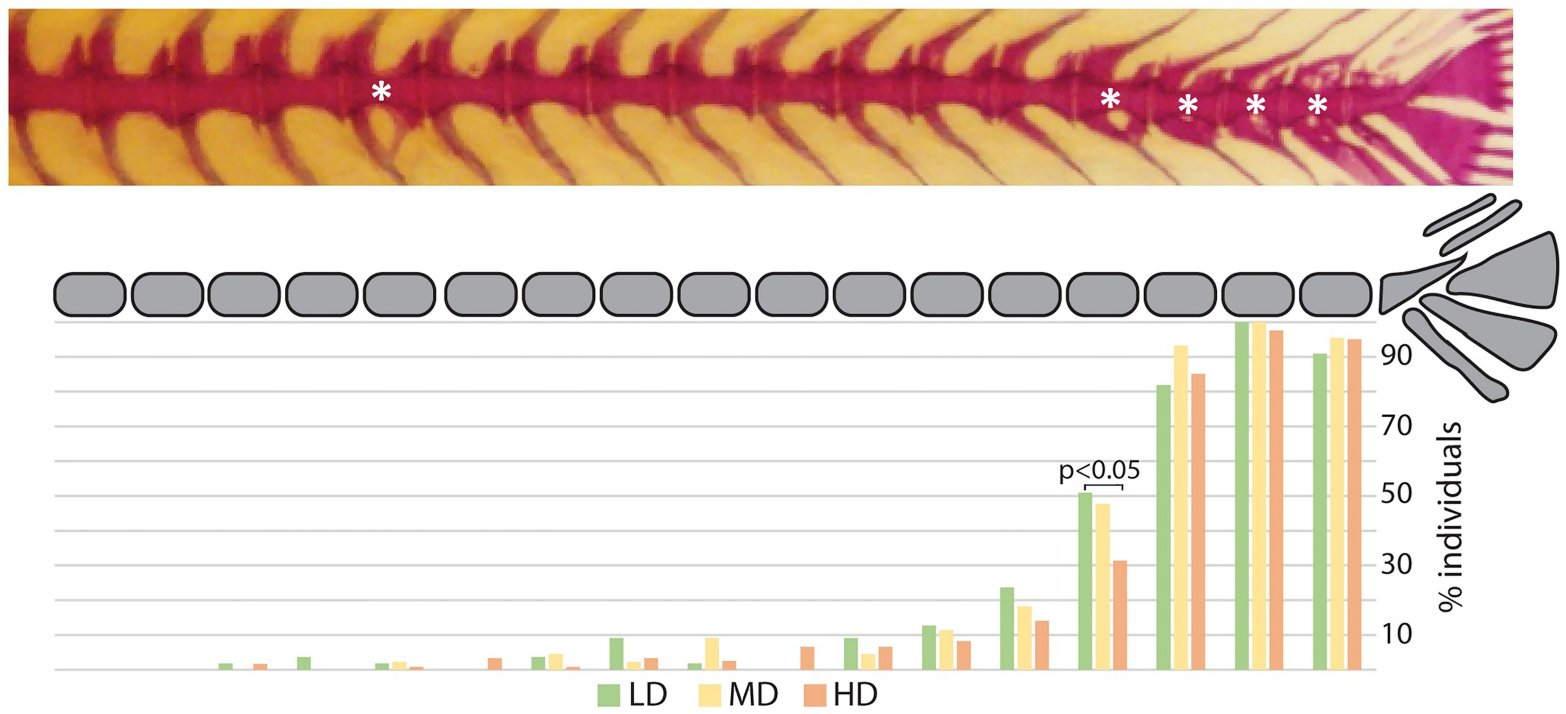
Hemal arch variability. Representative whole mount Alizarin red S-stained vertebral column of medaka (top). The asterisks indicate arches with connections between the ventral postzygapophysis and the hemal arch of the same vertebral body. The lower diagram reports a schematic representation of the vertebral column (caudal region and caudal complex) with the percentage of individuals showing a ventral connection for the corresponding vertebral body. Green: LD (low density); yellow: MD (medium density); red: HD (high density). “N-1” *chi-squared* test for proportions, followed by Bonferroni correction; p values are indicated for significant differences only.

The postcranial skeleton of medaka is composed of the vertebral column, paired fins, unpaired fins, and the endoskeletal fin support. The completely formed and mineralized vertebral column of medaka contains between 30 and 32 vertebrae, this includes the urostyle which is counted as a single unit. The vertebral column can be subdivided into 4 regions, as presented in **Figure 1**: cranial (2 vertebrae + basioccipital condyle), abdominal (9-11 vertebrae), caudal (14-16 vertebrae), and caudal complex (3-4 preural vertebrae plus urostyle). The last vertebra of the abdominal region that does not carry ribs and has open hemal arches is referred-to as a transitional region. The upper tips of the neural arches of the abdominal vertebrae are typically ‘fan-shaped’. Iwamatsu (45) reports that the upper tips of the spines of the 2^nd^-5^th^ vertebrae begin to develop this shape at 5.4 mm total length (TL), and that this process includes vertebrae 1-8 after the TL reaches 10 mm. A certain degree of variability is also observed in the shape of some hemal arches (**Figure 3**): i.e., the distal tip of ventral postzygapophysis connects to the arch by a bony bridge of variable thickness. This feature appears to be present in both the caudal and caudal complex vertebrae regardless of the rearing density but exhibits an increased occurrence when proceeding caudad. The position and frequency of these features is not significantly different between the experimental groups, except in the case of the last caudal vertebra, for which there are significant differences between LD and HD (p < 0.05).

**Figure 4:**
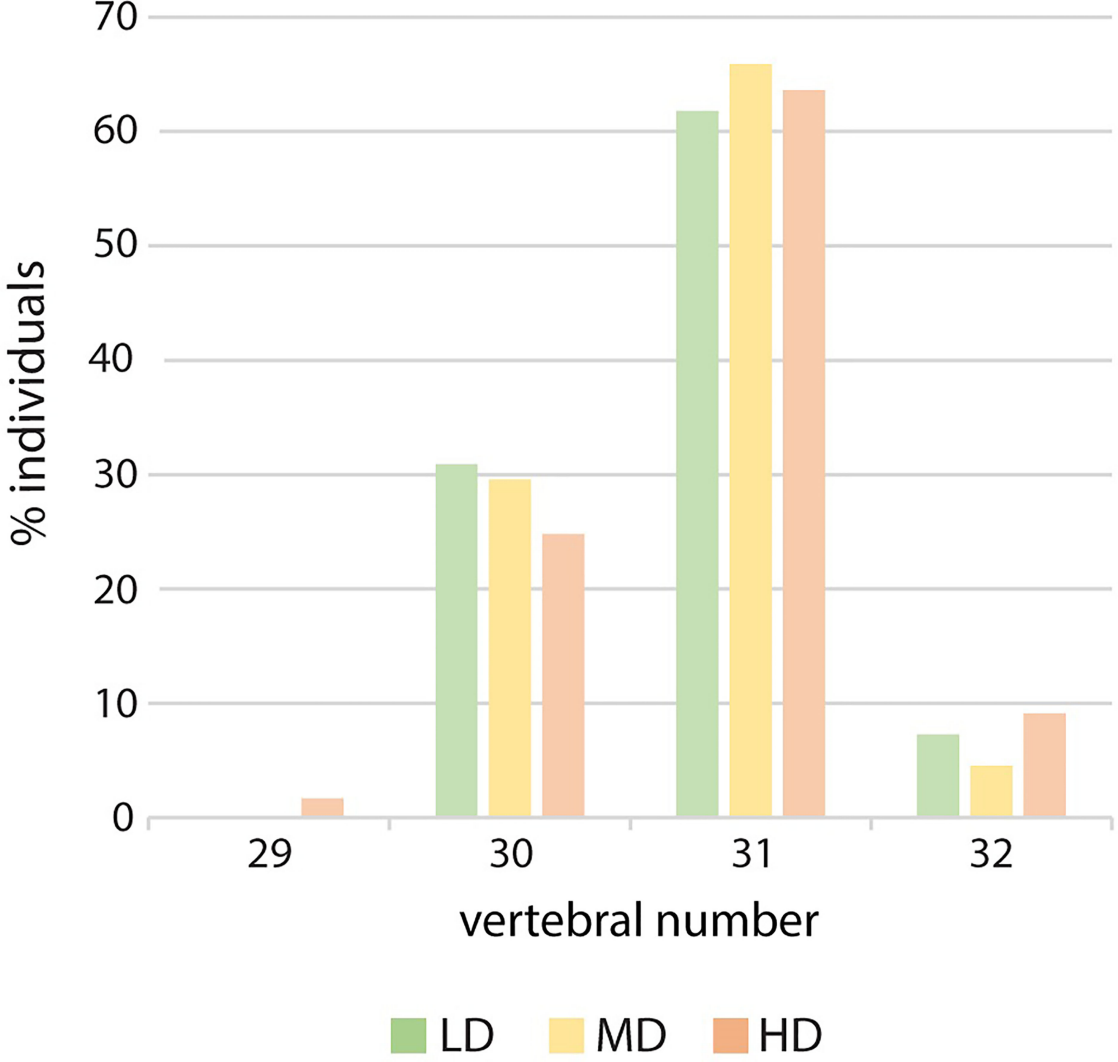
Vertebral number variation in relation to rearing density. Percentage of individuals with 29, 30, 31 or 32 vertebral centra. LD: low density; MD: medium density; HD: high density. *Chi-squared* to test the independence of three out of four vertebral numbers (30, 31 and 32). The large p-value (0.82) does not allow to reject the hypothesis of independence.

The meristic counts observed at the three experimental densities are reported in **Supplementary Table 3**. Counts that differ from those that have been previously published are: the numbers of dermal fin rays of the pectoral fins, 8-11 in this study vs. 9-10 or 9-11 in previous studies (22, 23, 26) and of the caudal fin, 6-7 inferior rays vs. 5-6 in a previous investigation (23). There are no remarkable differences in counts linked to density: a single HD medaka with 17 anal rays is observed and two HD individuals display only 29 vertebrae, due to vertebral centra fusions in the region of the caudal complex (**Figure 4**).

### Effects of Rearing Density on Mineralization of Skeletal Elements

**Figure 5:**
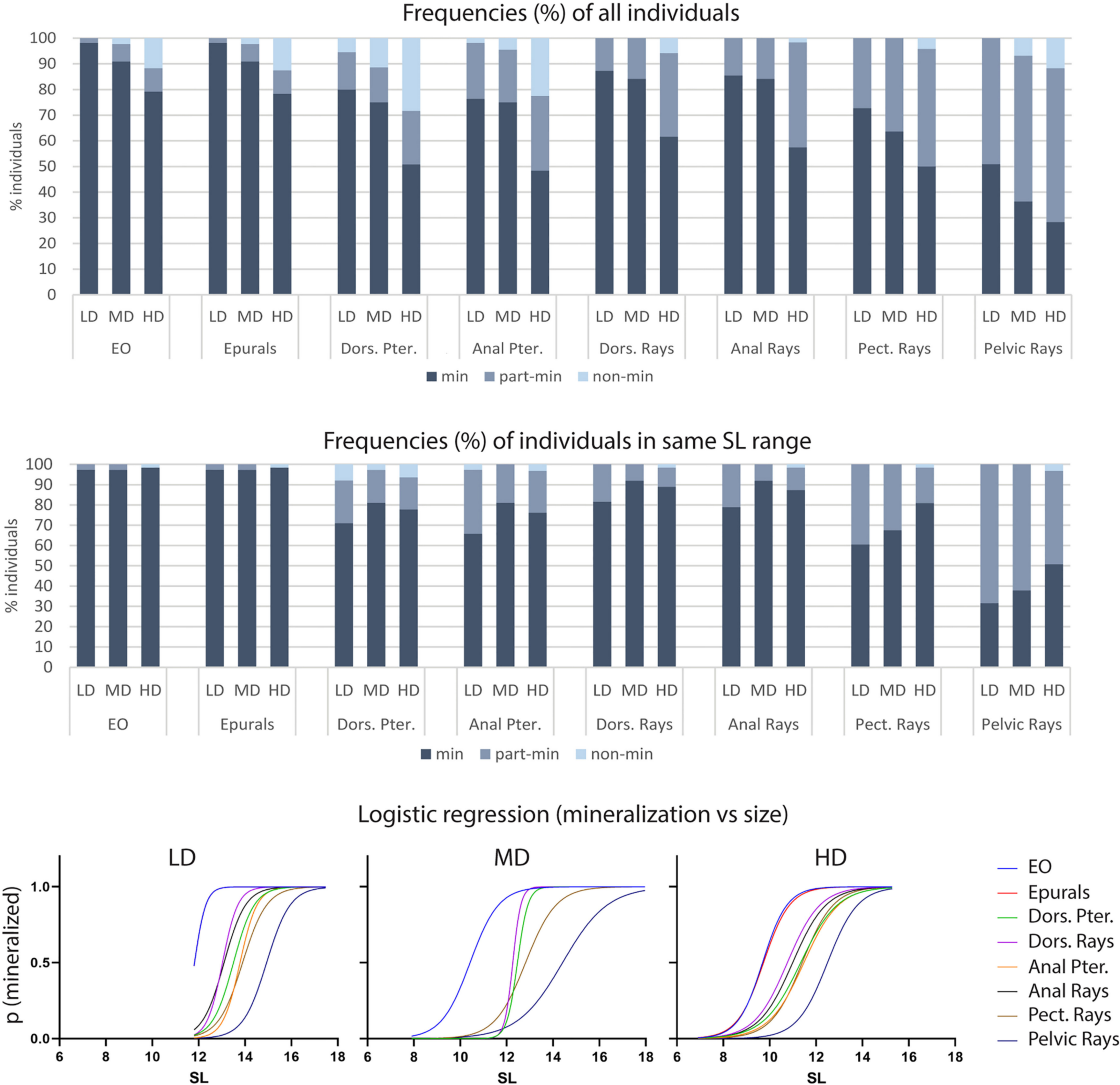
Mineralization of skeletal elements in relation to rearing density. The frequency (%) of individuals with fully (min), partially- (part-min), and non-mineralized (non-min) skeletal elements is reported for all individuals **(A)** as well as for individuals in the same size range **(B)**. **(A)** The percentage of fully mineralized skeletal elements decreases with the increasing rearing density. However, this density-related trend is lost when analyzing only individuals in the same size range **(B)**. **(C)** Logistic regression. The probability of identifying a mineralized skeletal element according to the standard length is reported for each density group: low (LD, left), medium (MD, center) and high (HD, right) density. An “S” shaped logistic function indicates a clear correlation between mineralization and size, whereas straight lines would indicate independency. Dors., dorsal; Pter., Pterygophore; Pect, pectoral; LD, low density; MD, medium density; HD, high density.

Bone formation involves the secretion of non-mineralized bone matrix while bone mineralization relates to the deposition of hydroxyapatite (16). We investigated if rearing density affects the mineralization of postcranial skeletal elements by examining the percentage of individuals with fully, partially-, and non-mineralized skeletal elements as indicated by the Alizarin red S staining. Vertebral centra and arches, parhypural (PH), modified hemal arch of PU2 (HPU2), and the dermal rays of the caudal fin are fully mineralized in all specimens, irrespective of the rearing density, therefore they are not shown in **Figure 5**. Conversely, the endoskeletal supports of all fins as well as the dermal fin rays of the dorsal, anal, pectoral and pelvic fins exhibit decreased levels of mineralization with increasing rearing density. The least mineralized skeletal element is the pelvic fin (LD: 51% of individuals with fully mineralized pelvic fin; MD: 36%; HD: 28%), followed by the pectoral, anal, dorsal, and caudal fin (**Figure 5A**). Taking into consideration that HD individuals are characterized by reduced size (**Figure 2**) compared to the other groups, the lower frequency of HD individuals with fully mineralized skeletal elements could be size-related. **Figure 5B** shows the mineralization levels of the individuals in the same size class (11.5-15.5 mm) for each density group. Although the HD condition appeared to be linked with reduced mineralization, these results were found to be confounding with the size of the fish. In fact, the stacked bar chart reveals the lack of a clear relationship between mineralization and rearing density. The dependency of the mineralization state on the size (SL) is statistically confirmed by a logistic regression analysis. The S-shape of the diagrams in **Figure 5C** signifies that the delay in mineralization for the partially- and non-mineralized elements is significantly dependent on the smaller SL (slope significantly different from 0 as tested with the Wald test and likelihood ratio test).

### Effects of Rearing Density on Skeletal Anomalies

**Figure 6:**
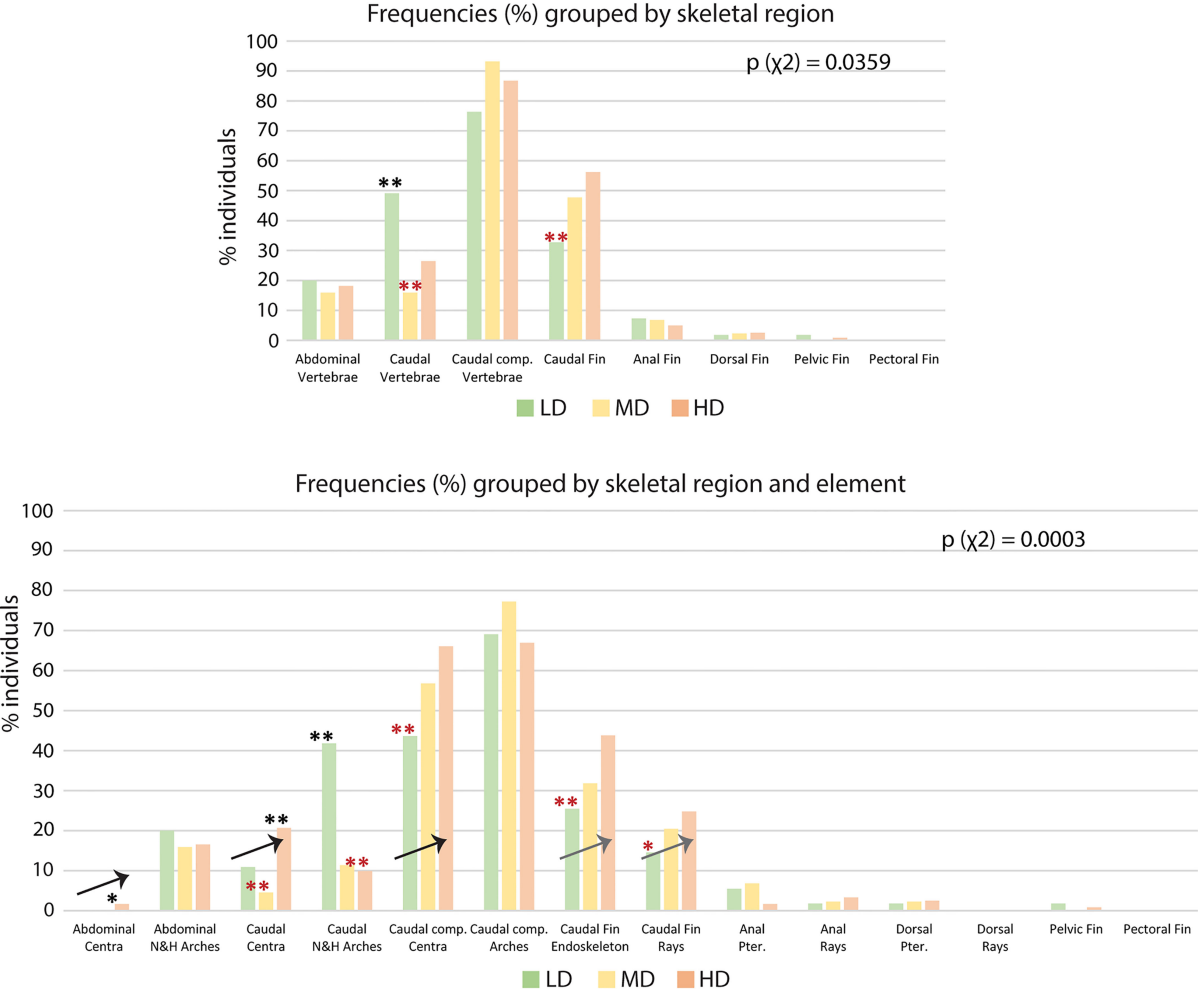
Skeletal anomalies linked to rearing densities. **(A)** Frequency (%) of individuals showing skeletal anomalies, grouped by region. The bars for the abdominal, caudal, and caudal complex vertebrae include anomalies of the centra and the arches. The data for the pectoral, pelvic, dorsal, anal, and caudal fin refer to anomalies of the dermal fin rays and the fin endoskeleton. **(B)** Frequency (%) of individuals showing skeletal anomalies, grouped by region and element. Arrows indicate the increase in the frequency of individuals with anomalies with increased rearing density. N&H arches, neural and hemal arches; Caudal comp., caudal complex; Pter., Pterygophore; LD, low density; MD, medium density; HD, high density. *Chi-squared* test followed by the analysis of the χ^2^ components, p-values are indicated on the respective graphs. Based on the analysis of the χ^2^ components, frequencies significantly higher or lower (*: p <0.05; **: p <0.01) than the expected values are indicated by a black or red asterisk, respectively.

The results shown in **Table 2** reveal the presence of at least one skeletal anomaly in nearly all of the individuals from each group (95% in LD; 98% in MD; 97% in HD). Interestingly, the frequency of individuals with axis or vertebral centra anomalies progressively increases from the LD- to the MD- and HD group (45%, 59% and 69%, respectively), resulting in significant differences between the LD and HD groups. In medaka, we do not observe extended axis deviations such as kyphosis, scoliosis, saddle-back syndrome, nor mismatched fusion of arches, which have been reported to occur in WT zebrafish (21). Contrarily, vertebral fractures are observed in this study although they have not been reported in wildtype zebrafish. **Supplementary Table 2** reports the frequencies (%) of individuals affected by each of the 62 types of detected anomalies. The analysis of skeletal anomalies in different regions of the postcranial skeleton shows that the caudal complex is most variable, irrespective of rearing density (**Figure 6A**). Within the caudal complex, preural vertebrae display the highest number of anomalies (LD: 76%; MD: 95%; HD: 87%) (**Figure 6A**). These are followed by caudal fin rays and endoskeleton (LD: 33%; MD: 50%; HD: 56%), the caudal (LD: 49%; MD: 16%; HD: 26%) vertebrae and abdominal vertebrae (LD: 24%; MD: 23%; HD: 18%). Cases of lordosis are rare: less than 1% of the individuals are affected. Dermal rays and endoskeletal elements of pectoral, pelvic, dorsal, and anal fins show low occurrences of anomalies. One LD medaka displays absent right pelvic fin rays and the pelvic fin endoskeleton. One HD medaka displays the same anomaly on the opposite lateral side.

Interestingly, the number of anomalies per malformed individual does not differ among the groups, while the frequency of individuals with severe anomalies (*viz.* axis and/or vertebral centra anomalies) increases at high rearing density. Localized lordotic, scoliotic, and kyphotic deviations, involving up to 4 vertebral bodies, are more frequently found in the caudal vertebrae and preurals, and most prominently found in the HD individuals (up to 6%). In general, neural and hemal arches are more variable, with no clear trend linked to the rearing density.

If we analyze anatomical skeletal elements made up of multiple developmental units, it is possible to further differentiate the response to rearing density; i.e., vertebral bodies (anatomical units) consist of vertebral centra and arches, which are distinct developmental modules (54). Likewise, each fin (anatomical unit) consists of multiple endoskeletal elements and dermal fin rays (developmental units). The caudal fin endoskeleton includes the PH, HPU2, EO, hypurals, and epurals. From this more detailed perspective (**Figure 6B**), vertebral centra, but not neural and hemal arches, show increased variability coinciding with increased density. The percentage of individuals with at least one vertebral centrum anomaly increases with density, i.e., from 11% (LD) to 21% (HD) in the caudal region and from 44% (LD) to 66% (HD) in the caudal complex. Complete vertebral centra fusions are observed only in preural centra, increasing from 2% in the LD and MD groups to 7% in the HD group. The caudal fin endoskeleton displays a similar trend, with anomalies increasing from 26% in the LD group to 44% in HD group. Likewise, the caudal fin ray anomalies increase from 14% in LD group animals to 25% in HD group animals. Concerning the dorsal and anal fins, **Figure 6B** reveals that the variability shown in **Figure 6A** relates to the pterygophores, rather than the dermal rays. Fusions in the preural centra are observed at different phases of fusion and modeling, in different individuals. This allows a detailed morphological and cellular investigation, as described in the following.

### Morphological and Histological Investigation of Vertebral Body Fusions in the Caudal Complex

**Figure 7:**
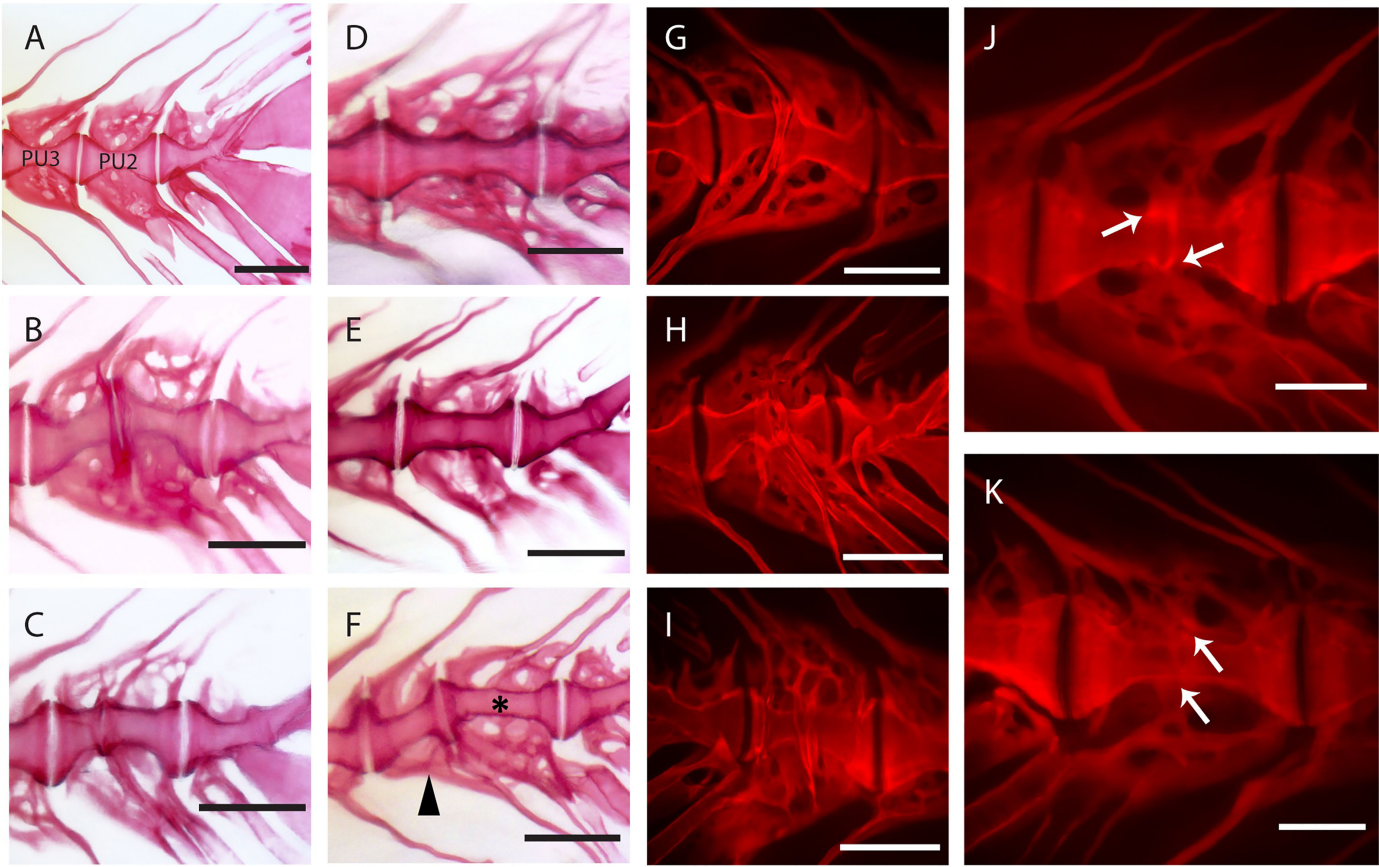
Static observations of fusing preural centra in medaka. **(A)** Caudal complex with non-deformed centra. **(B–K)** Different degrees of fusion between two preural centra. **(H, I)** and **(J, K)** show the right and left side of the same fusions, respectively. White arrows point to a clearly separated IVS in J and to a reshaped IVS in **(K)**. **(F)** Multiple fusion of preural centra: a vertebral body (arrowhead) is fusing to two previously fused and reshaped vertebrae (asterisk). PU: preural vertebra. Whole mount-staining with Alizarin red S. Scale bars = 400 µm **(A–I)**, 200 µm **(J–K)**

**Figure 8:**
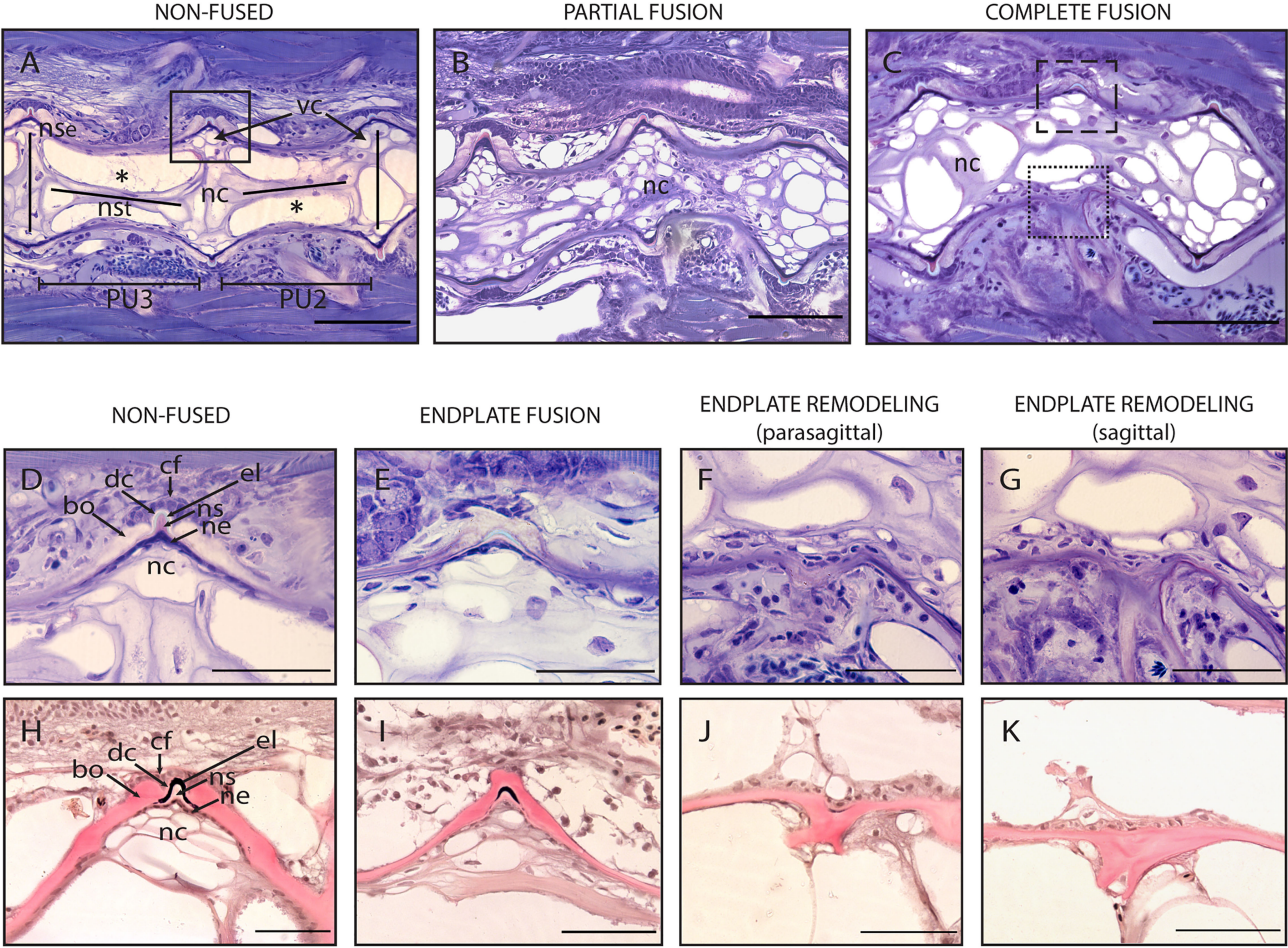
Non-fused and fused vertebral centra. **(A–C)** Overview of non-fused preural centra, partial fusion and complete fusion. Sagittal sections through preural 2 and 3 (PU2, PU3). **(D–K)** Vertebral body endplates at different stages of vertebral body fusion. **(A)** Two non-fused vertebral centra. The box indicates an intact intervertebral ligament, similar to the one magnified in **(D, H)**. **(B)** Partial fusion between two centra. The dorsal IVS is still intact, ventrally the vertebral body endplates are fused. **(C)** Complete fusion. Dorsally (dashed box), the endplates are fused, the intervertebral ligament is still present. Ventrally (dotted box), the fusion is complete, and the intervertebral ligament has disappeared. **(D, H)** Non-fused dorsal endplates with regular intervertebral ligaments. **(E, I)** Beginning of vertebral body fusion, dorsal. Vertebral body endplates are bridged by bone, intervertebral ligaments are still present. **(F, J)** Endplate remodeling (parasagittal plane), advanced vertebral body fusion. Only remnants of the outer elastin layer as a trace of the ventral intervertebral ligament can be recognized. **(G, K)** Endplate remodeling (sagittal plane), advanced vertebral body fusion as in F,J. The elastin layer and any other traces of the ventral intervertebral ligament are completely absent. **(A–G)** Toluidine Blue staining. **(H–K)** Elastin staining (Verhoeff). nc, notochord; *, notochord vacuoles; PU2, preural 2; PU3, preural 3; bo, bone; cf, collagen type I fiber bundles; dc, dense collagen type I matrix; el, elastin; nc, notochord cells; ne, notochord epithelium; ns, notochord sheath; nse, notochord septum; nst, nochord strand; vc, vacuolated chordocytes. Scale bars = 100 µm **(A–C)** and 50 µm **(D–K)**.

Observations carried out on whole mount-stained samples (**Figure 7**) and histological sections (**Figure 8**) seem to indicate that vertebral centra fusion likely starts by the fusion of the hemal arches of the two neighboring centra (**Figures 7B, C**). Normal (**Figures 7A**, **8A**) consecutive vertebrae are separated by regular intervertebral spaces (IVS) with intervertebral ligaments and notochord tissue. The detailed structure of a typical IVS is presented in **Figures 8D**, **8H**. Vacuolated notochord cells and the cells of the notochord epithelium are surrounded by a ligament composed of the notochord sheath, the outer elastin layer of the notochord sheath, and collagen type I fiber bundles that connect the vertebral body endplates (54). In what is interpreted as an early-stage fusion, hemal arches of the adjacent vertebrae are intertwined, and the dorsal intervertebral space and neural arches are still separated (**Figure 7B**). In the case of an advanced or complete fusion (**Figure 7E**), the dorsal intervertebral spaces and neural arches fuse (**Figures 7D–F**). In **Figures 7E, F**, it is possible to observe a vertebral fusion remodeled and reshaped into a normal (albeit elongated) centrum.

In addition to a ventral-dorsal asymmetry occurring during fusion and remodeling, we also observe some fusions that exhibit a left-right asymmetry in the vertebral centra shape (**Figures 7H–K**). In these fusions, the centra appear to be laterally deviated, as in a localized scoliosis involving only two vertebrae. Centra in a status of fusion show a clear separation on one lateral side and a completely fused and modeled centrum on the opposite lateral side (compare **Figure 7J** with **Figure 7K**, white arrows). The arches are not modeled and remain distinguishable. Fusions that involve more than two vertebral bodies are an uncommon observation (2 out of 12 specimens present complete or partial fusions, **Figure 7F**, asterisk and arrowhead).

To elucidate the cellular processes involved in vertebral body fusions, sagittal sections of normal and fused preural vertebrae 2 and 3 are analyzed. **Figure 8** shows histological observations that reflect the dorsal-ventral asymmetry observed on whole mount Alizarin red S-stained specimens. In a normal vertebral centrum at 40 dph, the vacuolated notochord cells and extracellular vacuoles are present. Dorsal and ventral IVS are connected by the notochord septum, septa of neighboring vertebral bodies are connected by the notochord strand (**Figure 8A**), a typical situation for a mature teleost notochord (54). The elements of the intervertebral ligament between preural 2 and preural 3 (**Figures 8A, D, H**) are regularly shaped and no alterations are observed. **Figures 8B, C** show sagittal sections of partially and completely fused vertebral bodies, respectively. In the partial fusion, the dorsal intervertebral space is still unaltered although ventrally, the two vertebral bodies appear completely fused (**Figure 8B**); and the components of the intervertebral ligament are preserved inside the bony bridge that connects the ventral vertebral body endplates. In completely fused vertebral bodies the dorsal endplates are also fused by a bony bridge (**Figures 8C, E, I**). At this stage, the elements of the ventral intervertebral ligament are no longer distinguishable, except traces of the notochords’ outer elastin layer (**Figure 8F, J**).

Based on these static observations of different degrees of vertebral fusion, we propose that the first step in the fusion process is the deposition of ectopic bone onto the IVS. This bone connects the endplates and bridges the IVS (**Figures 8E, I**). In this initial phase, the components of the intervertebral ligament, the collagen type-II based notochord sheath and its outer elastin layer are still present. The following stages, named as ‘endplate remodeling’, are shown in **Figures 8F** and **8J**, **Figures 8G** and **8K**. Parasagittal and sagittal sections reveal different levels of tissue reorganization: in the parasagittal plane (**Figures 8F, J**), small traces of elastin and collagen are detectable, but not in the sagittal plane (**Figures 8G, K**).

**Figure 9:**
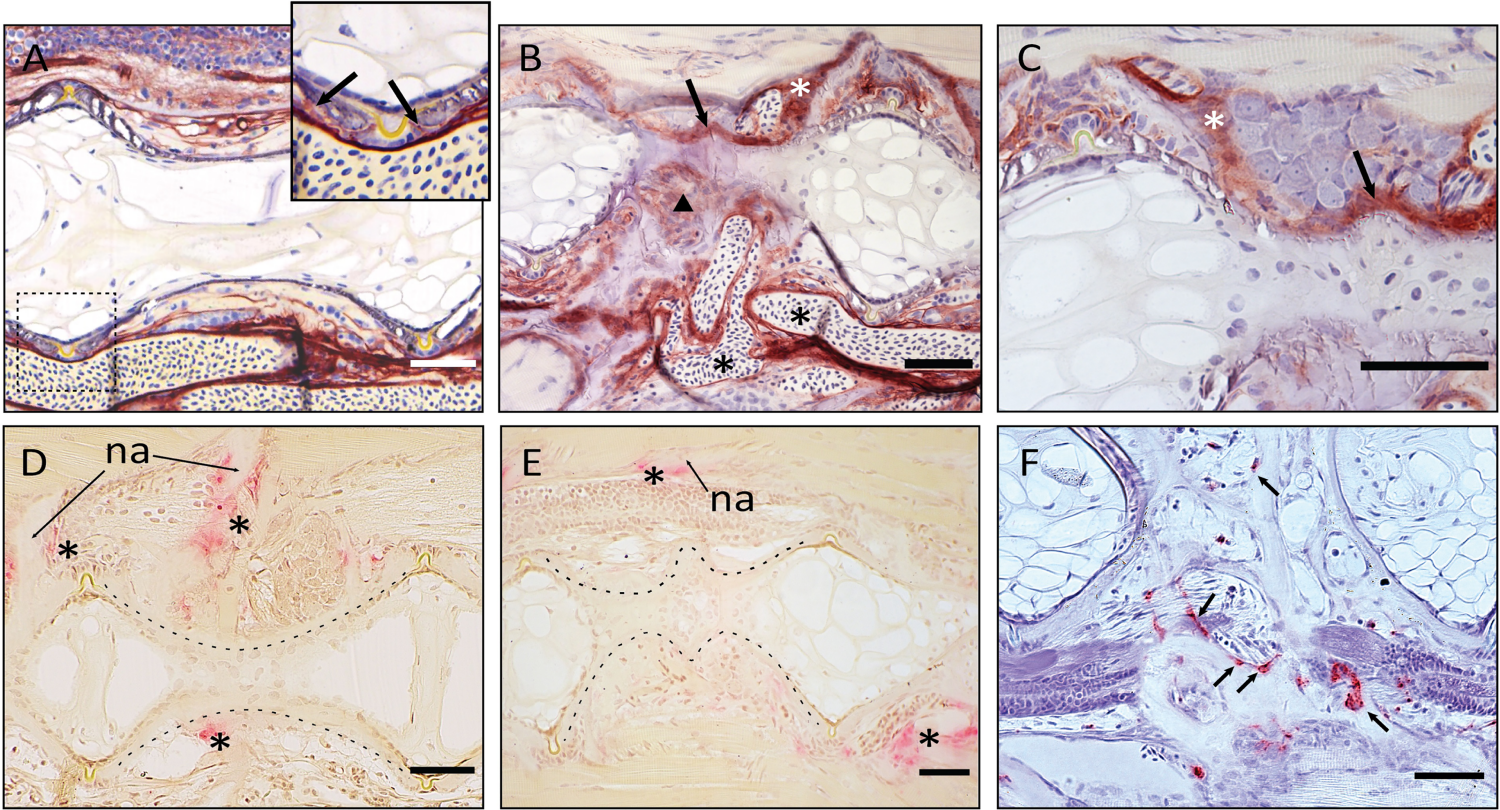
Demonstration of ALP and TRAP activity on the fused vertebral bodies. **(A–C)** ALP staining of vertebral bodies, sagittal sections, at the level of the intervertebral space. Dark red is indicative of ALP activity. **(A)** normal vertebral body. The enlarged picture shows the ventral intervertebral ligament in the dashed box. ALP is regularly expressed by osteoblasts, as indicated by the arrows. **(B)** Fused vertebral bodies. ALP is detected over the intervertebral space, connecting the fusing vertebral bodies (arrow), in the unorganized fusing hemal arches (arrowhead), as well as along the arches (white asterisk). Highly unorganized blood vessels are indicated by the black asterisks. ALP in endothelial cells of blood vessels should not be confused with osteoblast-secreted ALP. **(C)** Fusion centrum, at larger detail. ALP signal bridges neighboring vertebral bodies (arrow) and is detected along the fusing neural arches (white asterisk). **(D–F)** TRAP activity in fusing vertebral bodies, the dashed lines help visualizing the normal and fusing centra. **(D–E)** TRAP staining according to Witten et al. (44). Asterisks mark TRAP activity, detectable only at the endosteum of neural (asterisks above the centrum) and hemal (asterisk below the centrum) arches in control and fusing vertebral bodies. TRAP-positive osteoclasts are not detectable on the fusing vertebral centra **(E)**. na: neural arch. **(F)** TRAP staining according to Nemoto et al. (50). Parasagittal section of fusing vertebral bodies. TRAP-positive osteoclasts (arrows) are involved in the modeling of hemal arches after fusion has occurred. Scale bars = 50 µm.

The reshaping of fused vertebral bodies, particularly bone formation and bone resorption, are revealed by the activity of ALP (alkaline phosphatase) as a marker for osteoblasts and TRAP (tartrate-resistant acid phosphatase) as a marker for osteoclasts (**Figures 9A–F**, respectively). ALP is expressed by mature osteoblasts and can be visualized by enzyme histochemistry as a strong red staining. TRAP is a lysosomal osteoclast-specific enzyme that is secreted by osteoclasts into the cells’ subcellular space. Thus, TRAP not only labels bone resorbing cells but also sites of bone resorption. In a normal (i.e., non-fused) vertebral body, ALP is expressed by the osteoblasts on the growth zone of the vertebral body endplates (**Figure 9A**, arrows) and at the bone surfaces of the growing neural and hemal arches (**Figure 9C**, white asterisk). In fusing centra, ALP signal is detected on the surface of the fused IVS, thus bridging neighboring vertebral bodies (**Figure 9B**, arrow) and in unorganized bone structures that connect hemal arches (**Figure 9B**, arrowhead). The sections also highlight abundant vascularization in the region of the fusion (**Figure 9B**, black asterisks). In **Figures 9D–F**, TRAP can be identified based on a typical dark pink staining product. In a non-fused vertebral body, TRAP is visible at the endosteal bone surfaces of neural and hemal arches (**Figure 9D**, asterisks). At these locations, TRAP activity is required to facilitate the expansion of the arches during growth. In partially and completely fused vertebral bodies (**Figure 9E**), TRAP-positive osteoclasts are evidently involved in the reshaping of arches, individuated by the signal on the unorganized bone structures of fused hemal arches (**Figure 9F**). However, TRAP activity is absent on the surface of the fusing centra (**Figure 9E**).

## Discussion

This study analyzes the structure and variability of the postcranial skeleton in juvenile medaka raised at three different stocking densities. In particular, the focus is set on the effects of rearing density on growth, bone mineralization, meristic counts of skeletal elements, and skeletal anomalies. The results from this study could help distinguishing between skeletal variants that can occur in WT animals, anomalies related to laboratory rearing conditions and anomalies related to genetic strains. Thus, when screening for skeletal phenotypes resulting from gene editing, it is fundamental to take into account the rearing conditions (i.e., the influence of the environment), natural variability, skeletal phenotypic plasticity, and the genetic background along with species-specific peculiarities.

### The Response of the Skeleton to Rearing Density: Natural Variability or Malformations?

Almost all the observed medaka are affected by at least one skeletal anomaly (**Table 2**). Some of them appeared to be not significantly related to rearing density. For example, we observe variations in the hemal arches (**Figure 3**) that are possibly linked to SL rather than stocking density. This shape variability could be considered as a developmental feature that appears in larger medaka (for HD medaka: SL> 7.5 mm, median: 11.9 mm), as evidenced by the absence of significant differences in both the frequency and position of these features between the density groups. Generally, our data indicate that the frequency of individuals affected by anomalies of the arches and of all fins except the caudal fin, are not significantly dependent on rearing densities. Thus, these can be considered as natural variations. To discern which anomaly can be considered as natural variation and which is a deformation is difficult, to say the least. Following the definition of Mary West-Eberhard (19), “anomalies, or low-frequency discrete phenotypes, along with seemingly unpatterned variation called imprecision or noise, are often passed over in studies of variation, as if unusual variation were the enemy of insight” (19, page 205). Referred to as ‘minor skeletal variants’, numerous skeletal anomalies have been described in terrestrial mammals, including mice, small rodents, and humans (19, 55). In particular, in rodents, up to 98% of individuals showed at least one skeletal variant (56–59). These studies challenge the meaning of ‘normal’ because there are no ‘normals’ as such in nature (19). Likewise, also in some freshwater fish, minor skeletal variants have been described. Martini et al. (21) illustrates the existence of some skeletal variants (referred to as ‘background anomalies’) in wildtype zebrafish, whose occurrence is unaffected by the experimental conditions. Ferreri et al. (29) report that 87.2% of wild zebrafish display at least one skeletal anomaly, compared to 93.4% in the reared progeny of the same wild fish. In contrast, skeletal variability in marine species is rarely reported, except for fish sampled from polluted waters or aquaculture (60–62).

### The Effects of Rearing Density on the Skeleton of Medaka

The first clear outcome of this study is that rearing densities greater than 5 medaka/L produce significantly shorter (SL) juveniles, whose length progressively decreases with increasing densities. The observation of decreased length as a consequence of high density is in line with similar studies performed on gilthead seabream (*Sparus aurata*), zebrafish, rainbow trout (*Oncorhynchus mykiss*), and halibut (*Hippoglossus hippoglossus L.*) (21, 62–64). However, it is challenging to determine whether density is the only factor influencing the growth or if there are other confounding factors at play. In this study, feeding *ad libitum* three times per day was meant to avoid the development of size differences due to food shortage. However, the possible instauration of some “size-hierarchy” in crowded aquaria (65) could have led to competition between smaller and larger individuals, thus reducing the access to food for smaller individuals.

At 40 dph (SL range: 7-18 mm), the vertebral column of all animals in this study is completely mineralized. This corresponds to the observation of Iwamatsu (45) who described a fully mineralized vertebral column in medaka (d-rR strain, reared at 26°C) starting from a TL of 10 mm. As shown in **Figure 5**, the delay in mineralization of dermal fin rays and fin endoskeletal elements observed at higher densities is significantly related to the reduced length of HD animals and not to the HD condition *per se*.

In this study, the meristic counts of all elements are reported for a laboratory wildtype strain (**Supplementary Table 3**). Our data are in line with those reported by Parenti (22) and Roberts (23) for wild adult medaka sampled in various regions of Asia. The minor differences we found could be ascribed to particular traits from our laboratory strain, potential age differences (lower number of pectoral rays) or the possible influence of the natural environment (higher number of caudal rays). In this study, we observe only minor differences between the density groups: the number of vertebral bodies and the number of anal fin rays. The presence of 29 instead of 30-32 vertebrae is found in two HD individuals, however this is likely due to the complete fusion of preural 2 and 3. MD juveniles show higher median values for anal fin rays and pterygophores compared to LD and HD animals. A single HD medaka shows a minimum number of 17 anal rays, but this could be related to the small size of the individual (SL: 8 mm). Ali and Lindsey (25) report that in medaka, caudal and anal ray counts are the most susceptible to changes in response to varying environmental factors up to hatching, however in our study newly-hatched larvae are immediately subjected to different experimental conditions.

Similar to meristic counts that are found to have little variance, the percentage of medaka with at least one skeletal anomaly does not significantly vary between the three experimented densities (95% LD-, 98% MD-, and 97% HD-juveniles), with the lowest incidence found in the LD group (**Table 2**). The presence of at least 95% of medaka affected by skeletal anomalies can be explained by the methodology used. Our detailed analysis considers any detectable minor variation in size and shape of skeletal elements, which implies that diverse anomaly types (i.e., bifid neural spine, kypho-lordosis, vertebrae fusions, or misshapen caudal extra ossicle) are listed in the calculation of the metrics, regardless of their severity and associated functional impairment. Coherently, even the malformation index (number of anomalies per deformed individual) does not vary between the three groups.

Interestingly, the frequency of individuals with axis or vertebral centra anomalies progressively increases from the LD- to the MD- and HD group (45%, 59% and 69%, respectively; **Table 2**), with significant differences between the LD and HD group. Likewise, vertebral body anomalies and axis deviations are predominant in laboratory zebrafish reared at high density and gilthead seabream under intensive farming conditions (21, 66). Therefore, these anomalies could be considered as density-enhanced deformities.

In this study it was observed that the frequency of vertebral centra anomalies increases from cranial to caudal in all experimental groups. This pattern is also observed in both wild and reared zebrafish (29). In contrast, anomalies of neural and hemal arches in medaka do not show regional differences but generally display a higher degree of variability, regardless of the rearing density. In zebrafish, neural and hemal arches are also highly variable, affecting up to 50% of wild zebrafish. In captivity, this percentage can increase up to 80% (29) but Martini *et al.* (21) report that arch anomalies in the caudal complex increase at high densities. Morphological investigations on zebrafish or medaka mutants often highlight the independent response of vertebral centra and arches (15, 67–69).

Concerning the anomalies of paired and unpaired fins, this investigation identifies the caudal fin as the most variable and responsive to the tested densities. It is noteworthy that two medaka individuals (one from LD and one from HD group) lack the right or left pelvic fin rays and the basipterygium.

### Rearing Density Increases Variation in Caudal and Caudal Complex Vertebrae and Caudal Fin Endoskeleton

The caudal complex vertebrae and the caudal fin endoskeleton of teleosts display a large degree of natural variation (70, 71). The results from this study show that increasing stocking density has an additional effect on the frequency of anomalies in the caudal and caudal complex vertebrae. Behavioral studies on rainbow trout demonstrated that high stocking density has a significant effect on several parameters, including swimming activity, oxygen consumption and muscular activity, compared to low rearing densities, with substantial changes in the swimming trajectories and the space utilization (46, 72). In zebrafish and other carp fishes (Cyprinids), pheromone release yield alarm reactions that stimulate agitated swimming and abrupt movements (73). Altered behavioral patterns involves burst swimming, circling, jumping and erratic movements, as a response to aggressive individuals (74). It could be hypothesized that such behavioral mechanisms are in place when zebrafish or medaka are reared at high density. During swimming, the vertebral column of fish flexes laterally. An *in vivo* x-ray motion analysis on striped bass (*Morone saxatilis)* reveals regional differences in lateral bending throughout the startle response (escape behavior), which results in large body bending generating vertebral rotations and translations. The study unveils greater bending in the caudal region, with the maximum attained angle in the caudal complex (75). Imaging of lateral displacement and curvature profiles during slow swimming and fast startle response in zebrafish reveal changes in the body curvature and strain distribution. During burst swimming the curvature of the caudal region increases, compared to a stiff head and abdominal region (76). Therefore, increased burst swimming due to the interactions between more individuals in high density could elicit greater mechanical loading on the caudal vertebrae. Mechanical stress is a fundamental player in bone formation and mineralization. Mechanical strain, primarily exerted through muscular activity, is required to maintain bone mass and reshape the bony structures (77–79). In zebrafish, swim-training during early development accelerates both perichondral and intramembranous bone formation (80). In adult zebrafish and medaka, daily sessions of physical training increase bone formation and mineralization, thereby promoting a healthy skeletal development (14, 81). In contrast, exhaustive training is shown to induce lordosis in zebrafish and seabass *Dicentrarchus labrax* (82, 83). Still, hemal lordosis has a great recovery potential in zebrafish juveniles: when transferred from a laminar flow to “static” water, over 90% of the lordotic individuals resume their straight vertebral column after one week (84). In Atlantic salmon (*Salmo salar*), altered mechanical load has been related to anterior-posterior compression of the vertebral column. The underlying hypothesis is a possible transformation of the bone growth zones and the concomitant replacement of the intervertebral notochord tissue by cartilaginous tissues (85). In light of all this, it can be proposed that distorted swimming resulting from high density rearing affects muscular activity and can possibly increase anomalies with a significant impact on the skeletal phenotypes.

### Fusion of Preural Centra: Insights Into the Formation and Remodeling Mechanisms of Acellular Bone

Vertebral body fusions can have different etiologies: they can be the result of injuries, infections (86), or abnormal posture. They can have unknown origins but they are also part of normal ontogenetic or phylogenetic processes. Non-pathological fusion of vertebrae is recorded in numerous extant and extinct vertebrates (87–90): the best-known examples are fusion of sacral vertebrae in tetrapods to provide support and rigidity (87, 88), and fusion of vertebrae in birds and oviraptorid dinosaurs to provide locomotion (89, 90). In some teleosts, vertebral body fusions in the caudal complex are an ontogenetic step in the development of the caudal fin endoskeleton (37, 70, 71).

**Figure 10:**
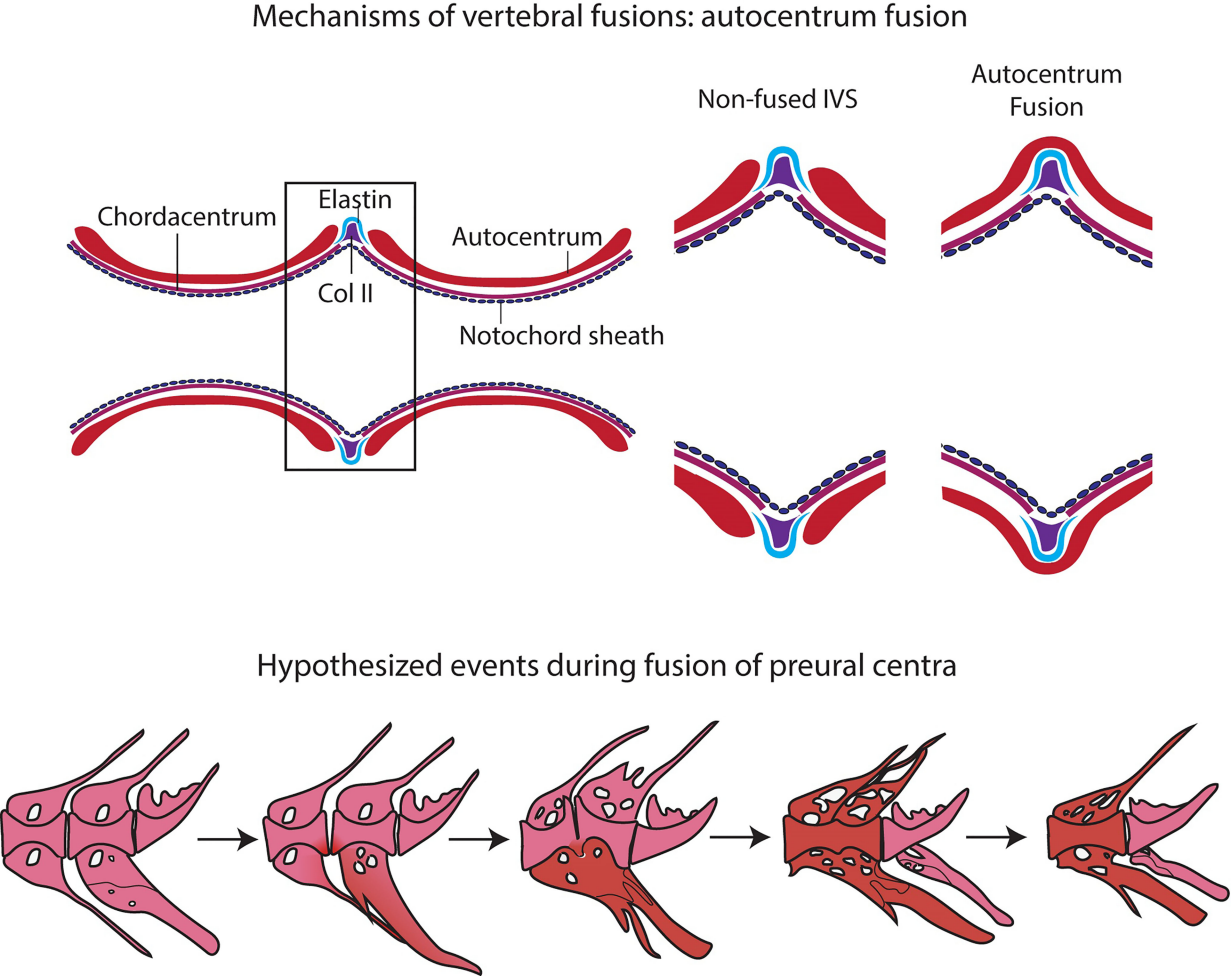
Events in vertebral body fusions. **(A)** In normal conditions, two consecutive vertebrae are separated by regular intervertebral spaces, with intervertebral ligament and notochord tissue. Schematic representation of autocentrum fusion: ectopic bone (red) is deposited over the IVS, with the intervertebral ligament still intact. **(B)** Hypothesized events during vertebral body fusion based on static observations. It can be hypothesized that the fusion starts at the ventral side by fusion of the hemal arches. The vertebral bodies consequently fuse ventrally first, with a regularly shaped intervertebral space on the dorsal side. Next, the vertebral bodies fuse also dorsally, and the fused centrum is reshaped into a normally looking (albeit elongated) vertebral centrum. In pink: normal; in red: fusing/fused.

Studies on zebrafish and Atlantic salmon have shown that fusions can occur *via* different processes, according to the stage of vertebral body development: (a) early fusions occur by continuous mineralization of the notochord sheath; (b) once bone has formed around the mineralized notochord sheath (autocentrum), fusions can occur by ectopic bone bridging the intervertebral space; (c) fusion of fully developed vertebral bodies typically involves the occurrence of ectopic cartilage located in the intervertebral space that is subsequently remodeled into bone (47, 91–93). Fusions of preural centra observed in this study are an example of the second fusion scenario (autocentrum fusion, **Figure 10A**).

The design of this study does not allow to follow the process of vertebral fusion in single individuals, but we observe a large number individuals in different stages of vertebral fusions. In view of the known fusion processes described in zebrafish and Atlantic salmon, it is possible to hypothesize about the sequence of events that lead to vertebral fusion in medaka (**Figure 10B**). The whole mount-staining reveals that the hemal arches fuse first and the ventral side of the fusion is remodeled and reshaped prior to the dorsal side. This leads to a dorsal-ventral asymmetry, which has been confirmed on a cellular level by the histological investigations. Assuming that the basis of the hemal arches fuses first, it could be speculated that the vertebral centra fuse as a secondary compensatory mechanism to the fusion of the arches. However, this latter hypothesis would require additional studies. The activity of the enzymes TRAP and ALP were investigated as proxies for osteoclasts and osteoblasts respectively, with the aim of gaining additional insights into the processes involved in the reshaping of the fused centra. In fact, remodeling and reshaping of teleost bone is based on the interplay between osteoblasts and osteoclasts (16), different from mammals where osteocytes regulate bone remodeling and osteoclast activity. In mammals, most osteoclasts are multinucleated giant cells that can easily be detected with standard histological procedures (94). Multinucleated osteoclasts can be absent in teleosts with acellular bone (as in medaka), yet acellular bone is resorbed, remodeled and responsive to mechanical load (14, 95, 96). According to Nemoto *et al.* (50) and Yu *et al.* (97), remodeling occurs in medaka by means of small and mononucleated osteoclasts. Indeed, acellular teleost bone must be remodeled in the frame of allometric growth, adaptation to mechanical load, and continuous tooth replacement (16, 48, 53, 95, 97–100). In growing teleosts, osteoclasts are present at endosteal bone surfaces enlarging skeletal elements, such as hemal and neural arches, but not on the vertebral centra (16, 44, 50, 53, 101). In our samples, partially and completely fused vertebral centra are negative for the osteoclast marker TRAP, even in locations that are evidently subjected to reshaping (**Figure 9**). Moreover, the lack of TRAP positive reversal lines renders the identification of previous remodeling events unfeasible (16). Possibly, at the vertebral centra, reshaping and fusion is only done by bone apposition. However, the absence of osteoclasts and bone remodeling demands further investigations.

### Knowledge Applied to Biomedical Research

Large scale screens for zebrafish mutants with dominant effect on morphology reveal several skeletal defects (102). Interestingly, many of these defects are also observed in individuals from natural zebrafish populations and wildtype laboratory zebrafish strains (21, 29). If ‘background’ or rearing density-related skeletal anomalies in wildtype medaka resemble defects generated in disease models is discussed below.

Sporadic mutations that can cause curvatures of the vertebral column naturally occur in teleosts. The *curveback* guppy occasionally presents a spinal deformity that resembles human idiopathic scoliosis (103), and wildtype zebrafish frequently display scoliotic vertebrae in the region of the caudal complex (21). Several zebrafish and medaka mutants exist with phenotypes that resemble scoliosis in humans. Zebrafish with a mutation in a kinesin family 6 protein gene *kif6* reveal a marked curvature of the abdominal and caudal spine (104). The *col8a1a* zebrafish mutant (*leviathan* mutant) is another model, associated with notochord defects and congenital vertebral malformations (105). Motile ciliary defects are a further factor promoting scoliosis in both zebrafish and medaka (106, 107). Thus, ciliary defects are correlated with a more extended skeletal phenotype, referred to as lordokyphosis, including left-right asymmetry of the body axis. These mutants are designated as *wavy* medaka (108, 109). Our investigation in medaka reveals that extended axis deviations are rare: we do not observe scoliosis or kyphosis, and cases of lordosis affect less than 1% of the individuals. However, more localized lordotic, scoliotic and kyphotic deviations, involving up to 4 vertebral bodies, are more frequent, most prominently in high density conditions.

Vertebral fusions, commonly observed in wildtype fish models, can also result from mutations. In mutants, fusions are mainly observed in the abdominal and in the caudal region. In medaka, disruption of vesicle trafficking from the Golgi to the ER by a nonsense mutation in *sec24d* gene induces vertebral centra fusions (110). A similar effect is observed as the result of the knockout of a gene coding for a metal ion transporter (*SLC39A8*) in zebrafish (111). *osx/sp7* mutations in juvenile zebrafish lead to low bone mineral density and intervertebral disk degeneration (112). *sp7-/-* adults reveal bent regions of the spine, compromised osteocyte lacunar profile, alterations in mineral density and altered collagen organization (113, 114). Interestingly, *osx* knockout in zebrafish is not linked to embryonic and post-embryonic defects in bone formation (113). According to our work, vertebral fusions in early medaka juveniles are detected only in the caudal complex. Although abdominal and caudal vertebrae are affected by skeletal anomalies, no fusion is observed in the abdominal or caudal region, in contrast to mutants.

Genetic mutations can also affect the patterning of the arches. In medaka, disruption of *sp7* leads to the absence of neural and hemal arches, although it does not affect formation of vertebral centra anlagen, i.e., the segmented mineralization of the notochord sheath, remains normal (115, 116). Likewise, the zebrafish *fused somites/tbx6 (fss)* mutants display normal vertebral centra and misplaced neural and hemal arches (67, 117). In addition to *tbx6*, Lleras-Forero *et al.* (118) mutated several genes involved in the zebrafish segmentation clock. Regardless of a disrupted segmentation clock, the notochord is normally segmented with regular chordacentra formation. On the contrary, the mutants display disrupted myotome boundaries and completely misplaced and deformed neural and hemal arches. Interestingly, we have no references about such severe deformities of the arches in wildtype medaka and observation in wildtype zebrafish are rare (21).

Concerning the unpaired fins (caudal, dorsal and anal), severe defects are rarely observed in wildtype individuals of zebrafish and medaka. In contrast, these are a prevalent characteristic of some severe mutant phenotypes. Henke and colleagues (102) report mutants with various degrees of phenotypes ranging from the reduction in ray number to the complete fin loss. Similarly, loss of function in the ectodysplasin (*eda*) and ectodysplasin receptor (*edar*) genes result in anomalies of the dermal skeleton (119). Anomalies of the paired fins (pectoral and pelvic) are more frequently observed in wild and wildtype zebrafish. Our investigation on wildtype medaka reveals that the pectoral fin is the least affected, whereas complete loss of pelvic fin rays and the supporting endoskeleton naturally occurred in two individuals.

In addition to considering the natural occurrence of skeletal anomalies in wild and wildtype individuals that might resemble defects resulting from genetic mutations, it is necessary to consider species-specific differences. The skeleton in medaka is overall more stable and less subject to anomalies and variations than zebrafish. According to this study, vertebral fusions are restricted to the caudal complex in medaka and are not detected in the caudal region. In contrast, partial and complete vertebral fusions are detected in wildtype zebrafish, however they are found less frequently in low-density rearing conditions (21). Likewise, axis deviations are rare in medaka, but more frequent in zebrafish, especially scoliosis affecting the preural vertebrae. A possible explanation to this reduced variability observed in medaka compared to zebrafish, is the smaller genome size but not the number of protein-coding genes (12, 13, 120). Variability is a fundamental process in development and evolution. However, as the organism complexity increases, the ability to alter a process or a character without effects on others processes and characters decreases (121). Organisms have the ability to reduce such variation. This tendency to buffer genetic and/or environmental perturbations is referred to as canalization (20, 122, 123). Thus, genetic or environmental canalization in advanced teleosts could explain the reduction in phenotypic plasticity of the skeleton.

## Data Availability Statement

The original contributions presented in the study are included in the article/**Supplementary Material**. Further inquiries can be directed to the corresponding author.

## Ethics Statement

The animal study was reviewed and approved by Italian Ministry of Health, approval N° 133/2021-PR.

## Author Contributions

CDB performed the experiments, analyzed samples and data, and wrote the first draft of the manuscript. CB contributed to conception and design of the study and to data analyses. PW contributed to histological and histoenzymatic investigations. ZD and AM collaborated during the experimental rearing and setup. MS contributed to the statistical evaluation of the data. All authors contributed to manuscript revision, read, and approved the submitted version.

## Funding

This project has received funding from the European Union’s Horizon 2020 research and innovation program under the Marie Skłodowska-Curie grant agreement No. 766347 - BIOMEDAQU.

## Conflict of Interest

The authors declare that the research was conducted in the absence of any commercial or financial relationships that could be construed as a potential conflict of interest.

## Publisher’s Note

All claims expressed in this article are solely those of the authors and do not necessarily represent those of their affiliated organizations, or those of the publisher, the editors and the reviewers. Any product that may be evaluated in this article, or claim that may be made by its manufacturer, is not guaranteed or endorsed by the publisher.
