# Supplementary material for: A Baseline for Skeletal Investigations in Medaka (Oryzias latipes): The Effects of Rearing Density on the Postcranial Phenotype
Source: Front Endocrinol (Lausanne). 2022 Jun 30;13:893699. doi: 10.3389/fendo.2022.893699 (PMC9281570; doi:10.3389/fendo.2022.893699)
Supplement: Supplementary file 1 [file Table_1.docx]

Supplementary Material

# Supplementary Tables

**Supplementary table 1 List of alphanumeric codes employed for the monitoring of skeletal anomalies in medaka.**

**Supplementary table 2 Relative frequencies (%) of individuals affected by each anomaly type.**

**Supplementary table 3 Variation in meristic counts for medaka reared at three experimental densities.** The number of vertebrae and elements of the caudal, pectoral, pelvic, dorsal and anal fins are reported for each experimental condition. Grey background indicates counts obtained from the available literature: either wild individuals (22, 23) or laboratory strains (26, 27). The asterisk * indicates data not reported as counts, but as ontogenetic description. In bold, values that differ from previously reported data. Basipteryg.: Basipterygium; Pterygoph.: Pterygophore; Mn: minimum; Mx: maximum; Md: median; LD: low density; MD: medium density; HD: high density.

**Supplementary table 1 List of alphanumeric codes employed for the monitoring of skeletal anomalies in medaka.**

| Region code | Skeletal element code | Anomaly code | Description |
| --- | --- | --- | --- |
| A  B  C  D  E  F  G  H  I |  |  | Cranial vertebrae  Abdominal vertebrae (carrying ribs and open hemal arches, without hemal spines)  Caudal vertebrae (with closed hemal spines)  Caudal complex vertebrae (preurals and ural vertebrae)  Pectoral fin  Anal fin  Caudal fin  Dorsal fin  Pelvic fin |
|  | 1 | lor  µK  µL  µLK | Lordosis, extended  Kyphosis, localized (up to 4 vertebral bodies)  Lordosis, localized (up to 4 vertebral bodies)  Lordo-kyphosis, localized (up to 4 vertebral bodies) |
|  | 2 | par  fus  def  elo/red  fra | Partial vertebral fusion  Complete vertebral fusion  Vertebral deformation  Marked vertebral elongation/reduction in length  Fractured vertebral body |
|  | 3 | def | Deformed urostyle |
|  | 4 | def  sup/abs  sup/abs hemi  sup arch  bif | Misshapen neural arch and/or spine  Supernumerary/absent neural arch  Supernumerary/absent neural hemiarch  Extra bony structure attached to a regularly shaped neural arch  Bifid (forked) neural spine (the right and the left spine don’t fuse) |
|  | 5 | def  sup/abs  sup/abs hemi  sup arch  bif | Misshapen hemal arch and/or spine  Supernumerary/absent hemal arch  Supernumerary/absent hemal hemiarch  Extra bony structure attached to a regularly shaped hemal arch  Bifid (forked) hemal spine (the right and the left spine don’t fuse) |
|  | 7 | def  sup/abs | Misshapen rib  Supernumerary/absent rib |
|  | 8 | def  sup/abs  fus | Deformed fin ray inner support  Supernumerary/absent fin ray inner support  Fusion among consecutive fin ray support elements |
|  | 9 | def S/I  sup/abs  fus | Deformed superior (S) / inferior (I) hypural  Supernumerary/ absent hypural  Fusion among consecutive hypurals |
|  | 10 | def  sup/abs  fus | Deformed parhypural  Supernumerary/absent parhypural  Fusion among parhypural and other fin support elements |
|  | 11 | def  sup/abs  fus | Deformed epural  Supernumerary/absent epural  Fusion among consecutive epurals |
|  | 12 | abs  asym  def  sup/abs  fus  shape | Absent fin  Asymmetric distribution of rays  Deformed ray  Supernumerary/absent ray  Fusion among consecutive rays  All the rays are deviated – deformed fin shape |
|  | 21 | def | Deformed extra caudal ossicle (EO) |

**Supplementary table 2 Relative frequencies (%) of individuals affected by each anomaly type.**

|  | **B1lor** | **B2def** | **B2fra** | **B4def** | **B4bif** | **B7def** | **B7abs** | **C1lor** | **C1µL** | **C2def** | **C2fra** | **C4def** | **C4bif** | **C4abs** | **C4sup** |
| --- | --- | --- | --- | --- | --- | --- | --- | --- | --- | --- | --- | --- | --- | --- | --- |
| **HD (%)** | 0.83 | 0.83 | 0.83 | 0.83 | 0.83 | 10.74 | 4.96 | 0.83 | 1.65 | 19.83 | 0.83 | 3.31 | 2.48 | 0.00 | 0.83 |
| **MD (%)** | 0.00 | 0.00 | 0.00 | 2.27 | 2.27 | 13.64 | 4.55 | 0.00 | 2.27 | 2.27 | 0.00 | 6.82 | 0.00 | 0.00 | 0.00 |
| **LD (%)** | 0.00 | 0.00 | 0.00 | 3.64 | 0.00 | 16.36 | 10.91 | 0.00 | 0.00 | 10.91 | 0.00 | 14.55 | 1.82 | 1.82 | 1.82 |

|  | **C4sup arch** | **C5def** | **C5bif** | **C5fus** | **C5sup arch** | **D1lor** | **D1µK** | **D1µL** | **D1µLK** | **D2par** | **D2fus** | **D2def** | **D2elo** | **D2red** | **D3def** |
| --- | --- | --- | --- | --- | --- | --- | --- | --- | --- | --- | --- | --- | --- | --- | --- |
| **HD (%)** | 0.00 | 3.31 | 0.83 | 0.00 | 1.65 | 0.83 | 5.79 | 3.31 | 0.00 | 0.00 | 6.61 | 41.32 | 1.65 | 30.58 | 4.96 |
| **MD (%)** | 0.00 | 2.27 | 0.00 | 0.00 | 2.27 | 0.00 | 0.00 | 4.55 | 4.55 | 0.00 | 2.27 | 45.45 | 0.00 | 25.00 | 2.27 |
| **LD (%)** | 1.82 | 10.91 | 0.00 | 1.82 | 12.73 | 0.00 | 1.82 | 3.64 | 0.00 | 3.64 | 1.82 | 20.00 | 0.00 | 23.64 | 7.27 |

|  | **D4def** | **D4bif** | **D4abs** | **D4abs hemi** | **D4sup** | **D4sup hemi** | **D4sup arch** | **D5def** | **D5bif** | **D5abs** | **D5fus** | **D5sup** | **D5sup hemi** | **D5sup arch** | **G9**  **def S** |
| --- | --- | --- | --- | --- | --- | --- | --- | --- | --- | --- | --- | --- | --- | --- | --- |
| **HD (%)** | 32.23 | 18.18 | 6.61 | 4.96 | 9.09 | 23.14 | 3.31 | 9.92 | 3.31 | 0.83 | 5.79 | 9.09 | 1.65 | 1.65 | 1.65 |
| **MD (%)** | 27.27 | 34.09 | 13.64 | 0.00 | 25.00 | 13.64 | 2.27 | 11.36 | 4.55 | 0.00 | 2.27 | 4.55 | 2.27 | 0.00 | 0.00 |
| **LD (%)** | 21.82 | 34.55 | 7.27 | 9.09 | 1.82 | 12.73 | 1.82 | 9.09 | 0.00 | 0.00 | 7.27 | 7.27 | 1.82 | 3.64 | 1.82 |

|  | **G9**  **def I** | **G10 def** | **G11**  **abs** | **G11 fus** | **G11**  **def** | **G11**  **sup** | **G21 abs** | **G21 sup** | **G21 def** | **G22 def** | **G12**  **def** | **G12**  **asym** | **G12**  **shape** | **F8**  **def** | **F12**  **def** | **H8**  **def** | **I12**  **abs** |
| --- | --- | --- | --- | --- | --- | --- | --- | --- | --- | --- | --- | --- | --- | --- | --- | --- | --- |
| **HD (%)** | 6.61 | 6.61 | 0.00 | 2.48 | 3.31 | 4.13 | 0.00 | 19.83 | 11.57 | 21.49 | 6.61 | 10.74 | 7.44 | 3.31 | 1.65 | 2.48 | 0.83 |
| **MD (%)** | 4.55 | 2.27 | 0.00 | 0.00 | 2.27 | 4.55 | 0.00 | 15.91 | 9.09 | 9.09 | 9.09 | 9.09 | 4.55 | 2.27 | 6.82 | 2.27 | 0.00 |
| **LD (%)** | 1.82 | 1.82 | 1.82 | 1.82 | 1.82 | 3.64 | 1.82 | 14.55 | 9.09 | 5.45 | 3.64 | 7.27 | 3.64 | 1.82 | 5.45 | 1.82 | 1.82 |

**Supplementary table 3 Variation in meristic counts for medaka reared at three experimental densities.**

|  | **Vertebrae** | | | **Epurals** | | | **Hypurals** | | | **Caudal Fin** | | | | | | **Pectoral Fin** | | | | | | | | | **Pelvic Fin** | | | | | | | | | **Dorsal Fin** | | | | | | **Anal Fin** | | | | | |
| --- | --- | --- | --- | --- | --- | --- | --- | --- | --- | --- | --- | --- | --- | --- | --- | --- | --- | --- | --- | --- | --- | --- | --- | --- | --- | --- | --- | --- | --- | --- | --- | --- | --- | --- | --- | --- | --- | --- | --- | --- | --- | --- | --- | --- | --- |
|  |  | | |  | | |  | | | **Superior**  **Rays** | | | **Inferior**  **Rays** | | | **Radials** | | | **Right**  **Rays** | | | **Left**  **Rays** | | | **Basipteryg.** | | | **Right**  **Rays** | | | **Left**  **Rays** | | | **Pterygoph.** | | | **Rays** | | | **Pterygoph.** | | | **Rays** | | |
|  | Mn | Mx | Md | Mn | Mx | Md | Mn | Mx | Md | Mn | Mx | Md | Mn | Mx | Md | Mn | Mx | Md | Mn | Mx | Md | Mn | Mx | Md | Mn | Mx | Md | Mn | Mx | Md | Mn | Mx | Md | Mn | Mx | Md | Mn | Mx | Md | Mn | Mx | Md | Mn | Mx | Md |
| *Roberts (1998)* | 30 | 31 | - | - | - | 2 | - | - | - | 5 | 6 | - | 5 | 6 | - | - | - | - | 9 | 10 | - | 9 | 10 | - | - | - | - | 6 | 6 | - | 6 | 6 | - | - | - | - | 6 | 7 | - | - | - | - | 18 | 20 | - |
| *Parenti*  *(2008)* | 27 | 32 | - | - | - | 2 | - | - | - | - | - | - | - | - | - | - | - | - | 9 | 11 | - | 9 | 11 | - | - | - | - | 5 | 7 | - | 5 | 7 | - | - | - | - | 5 | 7 | - | - | - | - | 17 | 22 | - |
| *Iwamatsu (2013)** | - | - | - | - | - | - | - | - | - | - | - | - | - | - | - | - | - | 4 | - | - | - | - | - | - | - | - | - | - | - | - | - | - | - | - | - | - | - | - | 6 | - | - | - | - | - | - |
| *Iwamatsu (2003)** | - | - | - | - | - | - | - | - | - | 20 | | | | | | - | - | - | 9 | 10 | - | 9 | 10 | - | - | - | - | 5 | 7 | - | 5 | 7 | - | - | - | - | 5 | 6 | - | - | - | - | 17 | 18 | - |
| LD | 30 | 32 | 31 | 2 | 3 | **2** | 2 | 2 | 2 | 5 | 6 | 6 | **7** | **7** | 7 | 4 | 4 | 4 | **8** | 11 | 10 | **8** | 11 | 10 | 2 | 2 | 2 | 5 | 6 | 6 | 5 | 6 | 6 | 5 | 7 | 6 | 5 | 7 | 6 | 17 | 20 | 18 | 18 | 21 | 19 |
| MD | 30 | 32 | 31 | 2 | 3 | **2** | 2 | 2 | 2 | 5 | 6 | 6 | **6** | **7** | 7 | 4 | 4 | 4 | **8** | 11 | 10 | **8** | 11 | 10 | 2 | 2 | 2 | 5 | 6 | 6 | 5 | 6 | 6 | 5 | 7 | 6 | 5 | 7 | 6 | 17 | 20 | 19 | 18 | 21 | 20 |
| HD | 29 | 32 | 31 | 2 | 3 | **2** | 2 | 2 | 2 | 5 | 6 | 6 | **6** | **7** | 7 | 4 | 4 | 4 | **8** | 11 | 10 | **8** | 11 | 10 | 2 | 2 | 2 | 5 | 6 | 6 | 5 | 6 | 6 | 5 | 7 | 6 | 5 | 7 | 6 | 17 | 20 | 18 | 17 | 21 | 19 |
